# PAR2‐mediated cellular senescence promotes inflammation and fibrosis in aging and chronic kidney disease

**DOI:** 10.1111/acel.14184

**Published:** 2024-04-30

**Authors:** Sugyeong Ha, Hyun Woo Kim, Kyung Mok Kim, Byeong Moo Kim, Jeongwon Kim, Minjung Son, Doyeon Kim, Mi‐Jeong Kim, Jian Yoo, Hak Sun Yu, Young‐Suk Jung, Jaewon Lee, Hae Young Chung, Ki Wung Chung

**Affiliations:** ^1^ Department of Pharmacy and Research Institute for Drug Development, College of Pharmacy Pusan National University Busan Korea; ^2^ Department of Parasitology and Tropical Medicine, School of Medicine Pusan National University Yangsan Korea

**Keywords:** aging, fatty acid oxidation, fibrosis, inflammation, PAR2, SASP, senescence

## Abstract

Cellular senescence contributes to inflammatory kidney disease via the secretion of inflammatory and profibrotic factors. Protease‐activating receptor 2 (PAR2) is a key regulator of inflammation in kidney diseases. However, the relationship between PAR2 and cellular senescence in kidney disease has not yet been described. In this study, we found that PAR2‐mediated metabolic changes in renal tubular epithelial cells induced cellular senescence and increased inflammatory responses. Using an aging and renal injury model, PAR2 expression was shown to be associated with cellular senescence. Under in vitro conditions in NRK52E cells, PAR2 activation induces tubular epithelial cell senescence and senescent cells showed defective fatty acid oxidation (FAO). Cpt1α inhibition showed similar senescent phenotype in the cells, implicating the important role of defective FAO in senescence. Finally, we subjected mice lacking PAR2 to aging and renal injury. PAR2‐deficient kidneys are protected from adenine‐ and cisplatin‐induced renal fibrosis and injury, respectively, by reducing senescence and inflammation. Moreover, kidneys lacking PAR2 exhibited reduced numbers of senescent cells and inflammation during aging. These findings offer fresh insights into the mechanisms underlying renal senescence and indicate that targeting PAR2 or FAO may be a promising therapeutic approach for managing kidney injury.

## Graphical abstract

This study uncovers the role of protease‐activating receptor 2 (PAR2) in promoting cellular senescence and inflammation in kidney disease. PAR2 activation induces metabolic changes leading to cellular senescence in renal tubular epithelial cells, highlighting its significance in renal pathophysiology. Mice lacking PAR2 exhibit reduced senescence and inflammation, suggesting PAR2 and fatty acid oxidation (FAO) as potential therapeutic targets for managing kidney injury.

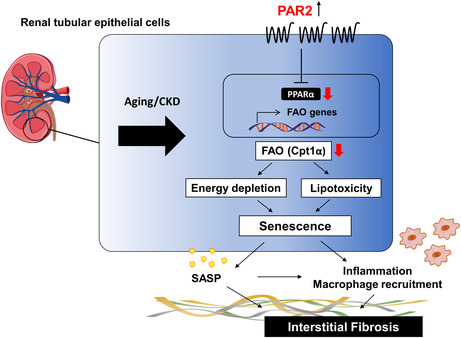

**Abbreviations**
ACOX1: acyl‐CoA oxidase 1
AD: adenine diet
AKI: acute kidney injury
ANOVA: analysis of variance
BUN: blood urea nitrogen
CKD: chronic kidney disease
CPT1: carnitine palmitoyltransferase 1
DAB: diaminobenzidine
DG: deoxy‐D‐glucose
DKD: diabetic kidney disease
ECM: extracellular matrix
FAO: fatty acid oxidation
FBS: fetal bovine serum
ISH: in situ hybridizaition
KO: knockout
OCR: oxygen consumption rate
ORO: oil red O
PAR2: protease‐activating receptor 2
SASP: senescence‐associated secretory phenotype
SA‐β‐gal: senescence‐associated β‐galactosidase
SD: Spraue–Dawley
SR: Sirius red
TG: triglyceride
WT: wild‐type

## 1 INTRODUCTION

The proportion of elderly people in the population is increasing steadily worldwide, accompanied by a continuous increase in life expectancy. Aging is a gradual process marked by the progressive decline in tissue function, resulting in the disruption of homeostasis and structural alterations in all organ systems (Lopez‐Otin et al., 2023). Among older adults, the prevalence of chronic kidney disease (CKD) is notably high, with over one third of individuals above the age of 70 years exhibiting moderate‐to‐severe CKD (Kovesdy, 2022). Kidney function declines with age, and is often accompanied by observable structural changes. Kidney fibrosis is characterized by excessive accumulation of extracellular matrix (ECM), infiltration of immune cells, and tubular atrophy (Huang et al., 2023). This is recognized as an important underlying pathologic process frequently observed in CKD development. However, the precise regulatory mechanisms underlying the treatment of renal fibrosis remain unclear.

Cellular senescence refers to a state of permanent cell‐cycle arrest characterized by heterogeneity and complexity. It is a natural biologic process that occurs as cells age or respond to various stressors, such as DNA damage, telomere shortening, oxidative stress, inflammation, mitochondrial damage, or oncogenic signaling (Kumari & Jat, 2021). The accumulation of senescent cells in tissues has been associated with age‐related disorders, including CKD (Huang et al., 2022). Senescent cells are characterized by a unique secretory phenotype called senescence‐associated secretory phenotype (SASP) (Xu et al., 2020). SASP include various secreted proteins, cytokines, growth factors, chemokines, and ECM‐modifying enzymes. Understanding the mechanisms and consequences of cellular senescence in kidney diseases is important for developing potential therapeutic strategies.

The kidneys are highly metabolically active organs that consume substantial amounts of energy. Despite only constituting a small percentage of the total body mass, the kidneys receive approximately 20% of the cardiac output, indicating their high energy demand (Duann & Lin, 2017). The kidneys possess an impressive ability to utilize lipid metabolites, enabling them to effectively meet their high energy demands (Kang et al., 2015). The dysregulation of lipid metabolism is commonly observed in kidney diseases and can contribute to the development and progression of various renal disorders (Herman‐Edelstein et al., 2014; Kang et al., 2015). Impaired fatty acid oxidation (FAO) is another key feature commonly observed in diseased kidneys (Chung et al., 2018; Chung, Dhillon, et al., 2019; Chung, Kim, et al., 2019; Kang et al., 2015). Reduced expression or activity of enzymes involved in FAO, such as PPARα, carnitine palmitoyltransferase I (CPT1), can hinder the breakdown of fatty acids and limit their utilization as an energy source (Miguel et al., 2021). Reduced FAO in kidneys has been associated with the induction of cell death or dedifferentiation, resulting in tubule atrophy and fibrosis (Dhillon et al., 2021; Kang et al., 2015).

Protease‐activating receptor 2 (PAR2) is a member of the subfamily of G protein‐coupled receptors and is activated by the specific cleavage of its extracellular N‐terminus by trypsin and other serine proteases (Heuberger & Schuepbach, 2019). Upon activation, PAR2 initiates intracellular signaling pathways that promote the inflammatory response (Heuberger & Schuepbach, 2019). While the role of PAR2 in metabolism has not been as extensively studied, emerging evidence suggests that it potentially influences metabolic processes (Kagota et al., 2016). PAR2 activation has been implicated in lipid metabolism in several tissues (Kim, Kim, et al., 2021; Kim, Puranik, et al., 2021; Rana et al., 2019). PAR2 activation has also been linked to increased lipid accumulation in liver cells, suggesting its potential role in hepatic lipid metabolism and fatty liver disease (Rana et al., 2019).

PAR2 has been found to play a role in the development of kidney disease by promoting the production of inflammatory mediators in tubular epithelial cells (Ha et al., 2022). However, the involvement of PAR2 in renal tubule cell senescence remains unexplored. In this study, we investigated the role of PAR2 in cellular senescence using aging and renal injury models. By uncovering the mechanistic relationship between PAR2 activation, FAO dysregulation, and tubule cell senescence, our study highlights the potential contribution of PAR2 to the progression of age‐related kidney diseases and kidney injury.

## 2 MATERIALS AND METHODS

### 2.1 Animal studies

All animal experiments were performed according to protocols approved by the Pusan National University Institutional Animal Care and Use Committee (Approval No. PNU‐2022‐0238). Sprague–Dawley (SD) rats were purchased from Samtako (Osan, Korea). Wild‐type (WT) C57BL/6J mice were purchased from Hyochang Science (Daegu, Korea), and PAR2 knockout (KO) (B6.Cg‐F2rl1^tm1Mslb^/J) mice were purchased from Jackson Laboratory (Bar Harbor, ME, USA). Male and female SD rats aged 6 and 20 months were used to evaluate the effects of aging on renal fibrosis according to sex (*n* = 6–7). For the kidney disease model, male and female WT mice (14 weeks old) were fed a 0.25% adenine diet (AD) for 3 weeks (*n* = 6–7). To examine the role of the PAR2 pathway in renal fibrosis, male WT and PAR2 KO mice (8 weeks old) were fed 0.25% AD for 3 weeks (*n* = 5–8). To investigate the role of PAR2 in fibrosis and senescence during kidney aging, we randomly divided male WT and PAR2 KO mice into four groups: young (6 months) and aged (20 months) (*n* = 6–8). To investigate the role of PAR2 in cellular senescence in the kidney, WT and PAR2 KO mice (6 months old) were intraperitoneally injected with either vehicle or cisplatin (10 mg/kg) once every 2 weeks for 4 weeks (*n* = 4–6). All rat and mice were kept at 23 ± 2°C, humidity of 60 ± 5%, and on a 12:12 h light: dark cycle with free accessible tap water and food. At the end of each study, serum was collected for biochemical analysis, and the harvested kidneys were fixed in neutral buffered formalin for histochemical examination or frozen at −80°C for further biochemical experiments.

### 2.2 Cell culture and treatments

NRK52E rat renal tubular epithelial cells were obtained from ATCC (Manassas, VA, USA) and cultured in DMEM supplemented with 5% fetal bovine serum (FBS). The cells were incubated at 37°C in a 5% CO_2_. To examine the effects of PAR2 on cellular senescence, NRK52E cells were treated with SLIGRL‐NH_2_ (150 μM, HY‐P1308, MedChemExpress, Monmouth Junction, NJ, USA) for 72 h. In order to examine the impact of PAR2 on lipid metabolism, NRK52E cells were exposed to oleic acid (50 μM) with or without SLIGRL‐NH2 (150 μM) for 24 h. To evaluate the effects of lipid accumulation on cellular senescence, NRK52E cells were treated with etomoxir (HY‐50202, MedChemExpress), a CPT1α inhibitor, for 72 h. NRK52E cells were treated with cisplatin (10 μM) for 6 h and incubated with 2% FBS for 72 h. For cell culture experiments, at least three different experiments were performed to ensure reproducibility.

### 2.3 Determination of blood urea nitrogen level

Serum samples were collected by centrifugation at 2500 *g* for 20 min at 4°C. Blood urea nitrogen (BUN) levels were measured using a commercial assay kit from Shinyang Diagnostics (SICDIA L‐BUN, 1120171; Seoul, Korea), according to the manufacturer's instructions.

### 2.4 Protein extraction and western immunoblotting

All tubes, solutions, and centrifuges were maintained at 4°C during the protein extraction. Kidney tissues were lysed using ProEXTM CETi protein extract solution (Translab, Daejeon, Korea) with a protease inhibitor cocktail (GenDEPOT, Katy, TX, USA) according to the manufacturer's instructions. Cells were extracted using RIPA buffer (Cell Signaling, #9806) containing a protease inhibitor cocktail. Proteins from cell and tissue lysates were quantified using a BCA protein assay kit (Thermo Fisher Scientific, Waltham, MA, USA). Western blotting was performed as previously described with minor modifications (Yang et al., 2021). Antibodies used in this study are listed in Table S1.

### 2.5 RNA extraction and quantitative real‐time PCR analysis

Total RNA was extracted from tissues or cells using TRIzol reagent (Invitrogen, Carlsbad, CA, USA) following the manufacturer's protocol. Total RNA (2000 ng) was reverse‐transcribed into cDNA using SuPrimeScript RT‐PCR Premix (SR‐4100; GenDEPOT, Katy, TX, USA). qRT‐PCR was performed using SYBR Green Master Mix (Bioline, Taunton, MA, USA) on a CFX Connect System (Bio‐Rad). mRNA primers were designed using the Primer3Plus software. The primer sequences are listed in Table S2. The 2^−ΔΔCt^ method was used for the calculation of fold changes. *Gapdh* gene was served as a reference gene.

### 2.6 Histology and immunohistochemical staining

Paraffin‐embedded kidney sections (5 μm thickness) were prepared by a routine procedure and stained with hematoxylin and eosin (H&E) or Sirius red (SR, Vitrovivo, Rockville, MD, USA) as previously described. Images were captured using an microscope (LS30, Leam Solution, Seoul, Korea). The positive area (%) was assessed using ImageJ software. Paraffin‐embedded sections were incubated with the indicated primary antibody, anti‐PAR2 (sc‐13504, Santa Cruz Biotechnology; dilution 1:100). After blocking avidin and biotin using an Avidin/Biotin Blocking Kit (SP‐2001, Vector Lab), the sections were incubated with a biotinylated secondary antibody and visualized using diaminobenzidine (DAB) substrates. Sections were counterstained with Mayer's hematoxylin (30002; Muto Pure Chemicals, Japan). Images were captured under a microscope (LS30; Leam Solution, Seoul, Korea).

### 2.7 Immunofluorescence

Paraffin‐embedded kidney sections were subjected to immunofluorescence staining using the Vector Fluor Duet Immunofluorescence Double Labeling Kit (DK‐8818, Vector Laboratories), according to the manufacturer's instructions. Primary antibodies used was as follows: anti‐PAR2 (sc‐13,504, Santa Cruz), anti‐Vimentin (#5741S, Cell signaling), and anti‐CPT1α (ab128568, Abcam). After washing, the slides were incubated with the VectaFluor Duet Reagent (DyLight 488 Anti‐Rabbit IgG and DyLight 594 Anti‐Mouse IgG cocktail). The sections were mounted using Antifade Mounting Medium with DAPI (Vector Laboratories). All the images were captured using a fluorescence microscope.

### 2.8 Staining and quantification of lipids

To detect lipid accumulation in the kidneys, OCT‐embedded tissues were sectioned at 8 μm. The frozen sections were fixed and stained with Oil Red O (ORO) solution (Sigma‐Aldrich) for 10 min. After washing with distilled water, the sections were stained with hematoxylin for 2 min. To detect the intracellular lipid content, NRK52E cells were washed with PBS, fixed in 4% PFA for 15 min at room temperature, and then rinsed in PBS. The cells were incubated with ORO solution for 30 min, washed thrice with distilled water, and stained with hematoxylin for 2 min. The accumulation of lipid droplets in tissues and NRK52E cells was observed under a microscope. Cellular triglyceride (TG) content was quantified using a PicoSens Triglyceride Assay Kit (BM‐TGR‐100, BIOMAX, Seoul, Korea), following the manufacturer's protocol.

### 2.9 In situ hybridization

In situ hybridization (ISH) of fresh paraffin‐embedded kidney sections and NRK52E cells was performed using either the RNAscope 2.5 HD Reagent kit‐RED for single‐gene detection or the RNAscope 2.5 HD Duplex Detection Kit (#322350 and #322436, Advanced Cell Diagnostics, Hayward, CA, USA) for dual gene detection, in accordance with the manufacturer's protocol. The following probes were used: Mm‐*Cdkn1a* (#408551‐C2), Mm‐*Ccl2* (#311791), Mm‐*F2rl1* (#417541), Mm‐*Col1a1* (#319371‐C2), Mm‐*Cpt1a* (#443071), and Mm‐*Emr1* (#317969‐C2). Images were captured using a microscope and quantification for positive area were calculated using ImageJ software.

### 2.10 Senescence‐associated β‐galactosidase assay

NRK52E cells and frozen kidney sections (8 μm) were processed to detect SA‐β‐gal activity using a commercial senescence‐associated β‐galactosidase (SA‐β‐gal) staining kit (ab102534; Abcam, Cambridge, United Kingdom) according to the manufacturer's instructions. Images were obtained using a microscope. Quantification of SA‐β‐gal‐positive cells were calculated using ImageJ software.

### 2.11 Determination of transcriptional activity

To detect PPARα transcriptional activity, cells were seeded and transfected with PPRE‐X3‐TK‐LUC plasmid (Dr. Christoper K. Glass at University of California, San Diego, CA, USA) and PPARα plasmid (Dr. Han Geuk Seo at Konkuk University, Seoul, Korea) using Lipofectamine 3000 (Invitrogen Corporation, San Diego, CA, USA) with Opti‐MEM (Gibco, Langley, Oklahoma) following the manufacturer's protocol. After 24 h of transfection, luciferase activity was measured using a One‐Glo Luciferase Assay System kit (Promega, Madison, WI, USA). The light intensity was detected using a luminescence plate reader (Berthold Technologies GmbH & Co., Germany).

### 2.12 Determination of cellular metabolic states

To determine the metabolic states of the PAR2‐activated cells, the oxygen consumption rate (OCR) was measured using a Seahorse Bioscience XFp Extracellular Flux Analyzer (Seahorse Bioscience, Billerica, MA, USA) following the manufacturer's instructions. NRK52E cells were seeded on a special plate at a density of 6 × 10^3^ cells/well. The cells were then incubated with SLIGRL‐NH2 (150 μM) for 24 h. The culture medium was replaced with the XF assay medium supplemented with 1 mM pyruvate, 2 mM L‐glutamine, and 10 mM glucose for the OCR assay. Basal OCR (pmol/min) was measured before the addition of rotenone (0.5 μM)/antimycin A (0.5 μM), followed by 50 mM 2‐deoxy‐D‐glucose (2‐DG) treatment. The OCR was normalized to the protein concentration. The lactate content of NRK52E cells was quantified using a PicoSens Lactate Assay Kit (BM‐LAC‐100, BIOMAX), following the manufacturer's protocol.

### 2.13 Quantification and statistical analysis

Graphing and statistical analyses were performed using GraphPad Prism 5 software (La Jolla, CA, USA). Unpaired Student's *t* test was used to analyze differences between two groups, and analysis of variance (ANOVA) was used to analyze intergroup differences. All data are presented as the mean values (±SEM). *P* <0.05 were considered statistically significant. Densitometry results of western blotting were quantified using ImageJ software.

## 3 RESULTS

### 3.1 Evaluation of age‐related renal changes in male and female SD rats

First, we used young and aged SD rat models (6 and 20 months old) to evaluate renal damage and the development of fibrosis (Figure S1A). The kidney weight/body weight ratio and serum BUN levels significantly increased in aged male rats, whereas no significant change was observed in aged female rats (Figure S1B,C). The extent of increase in kidney damage‐related gene and protein expression was higher in aged male kidneys than in aged female kidneys (Figure S1D,E). Histologic analysis revealed that male rat kidneys showed significant morphologic changes compared to the kidneys of female rats (Figure S1F). The expression of fibrosis‐related genes and proteins significantly increased in aged male rat kidneys, whereas no changes were detected in aged female rat kidneys (Figure S1G,H). Sirius red (SR) staining also showed significant development of fibrosis in aged male kidneys (Figure S1I). These findings indicate that male rat kidneys exhibit higher susceptibility to age‐related renal damage and fibrosis than female rat kidneys.

### 3.2 Tubule epithelial senescence is associated with renal aging and inflammation

**FIGURE 1:**
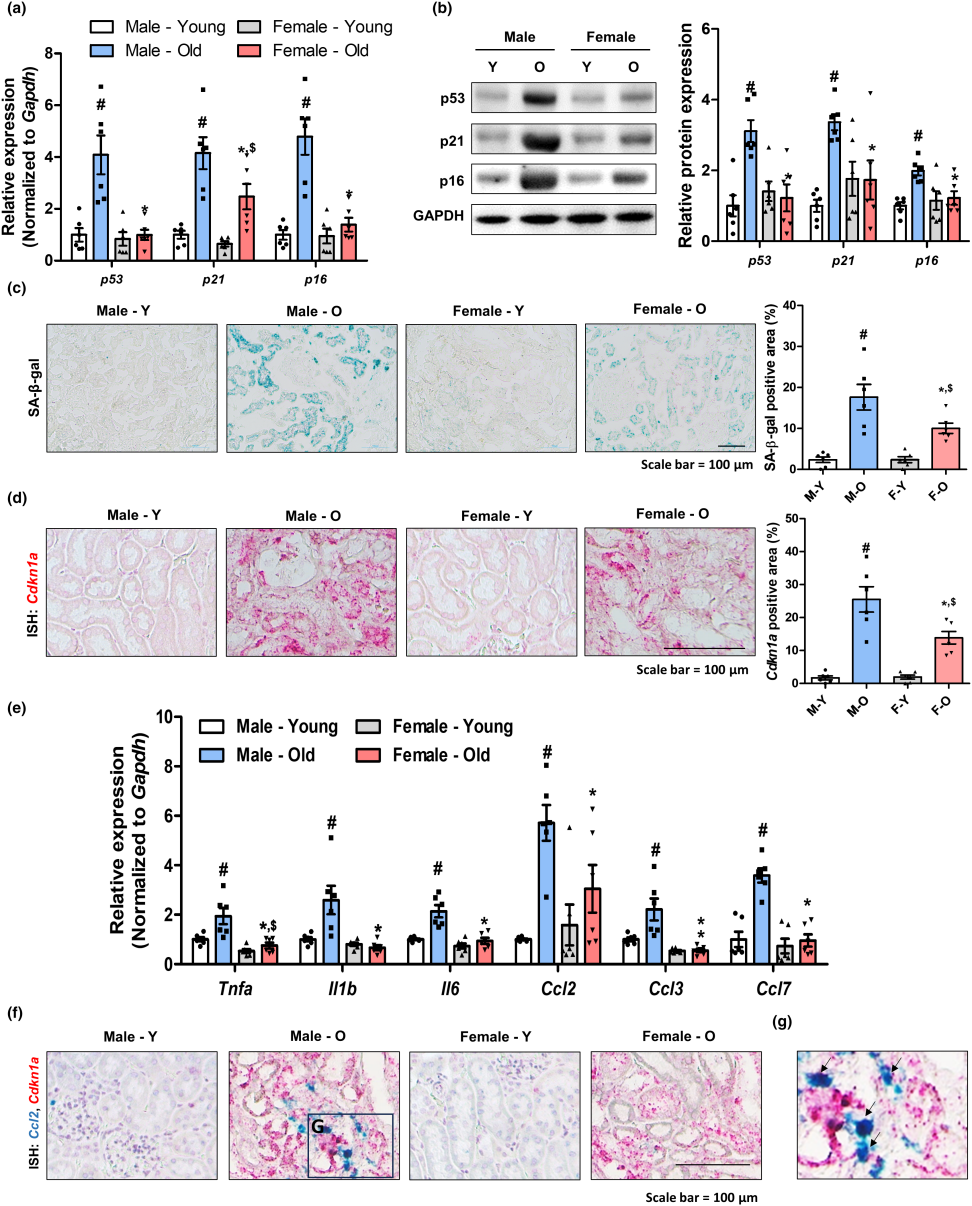
Tubule cell senescence is associated with increased renal injury in aged rat kidney. (a) Relative mRNA expression of *p16*, *p21*, and *p53*. #*p* < 0.05 versus male young rats. *p < 0.05 versus male aged rats. $*p* < 0.05 versus female young rats. (b) Representative western blots showing the renal expression of p16, p21, and p53 in four groups. GAPDH was used as internal control. Relative protein expressions were quantified using densitometry. #*p* < 0.05 versus male young rats. *p < 0.05 versus male aged rats. (c) Representative images of renal SA‐β‐gal staining in different groups. The area positive for SA‐β‐gal staining were quantified for each group. #*p* < 0.05 versus male young rats. *p < 0.05 versus male aged rats. $*p* < 0.05 versus female young rats. (d) Representative ISH images of *p21* (red) gene in four groups. The area positive for ISH staining were quantified for each group. #*p* < 0.05 versus male young rats. *p < 0.05 versus male aged rats. $*p* < 0.05 versus female young rats. (e) Relative mRNA expression of *Tnfa*, *Il6*, *Il1b*, *Ccl2*, *Ccl3*, and *Ccl7*. #*p* < 0.05 versus male young rats. *p < 0.05 versus male aged rats. $*p* < 0.05 versus female young rats. (f) Representative dual‐ISH images of *Ccl2* (green) and *Cdkn1a* (red) genes in different groups. (g) Magnified image of dual‐ISH staining from male aged rats. Arrows highlight cells positive for both *Ccl2* (green) and *Cdkn1a* (red).

Next, we investigated whether cellular senescence was associated with renal aging in aging models. Significantly elevated levels of senescence‐related genes and proteins were observed only in the kidneys of aged male rats (Figure 1a,b). Similarly, β‐galactosidase (SA‐β‐gal)‐positive signals were highly detectable in aged male kidney, especially in the tubule epithelial cells (Figure 1c). In situ hybridization (ISH) of the p21 gene (*Cdkn1a*) also showed that senescent cells were mostly present in the tubule epithelial region of aged male kidneys (Figure 1d). The expression levels of inflammation‐related were also significantly increased only in aged male kidneys (Figure 1e). Interestingly, *Cdkn1a*‐positive senescent tubule cells also expressed *Ccl2* when detected by dual ISH analysis, implying that senescent tubule cells contribute to the SASP phenotype in aged kidneys (Figure 1f,g). These data indicate that aging increases tubular epithelial senescence and contributes to increased inflammation in aged kidneys.

### 3.3 Tubule epithelial senescence is also detectable and contributes to inflammation in CKD mouse model

**FIGURE 2:**
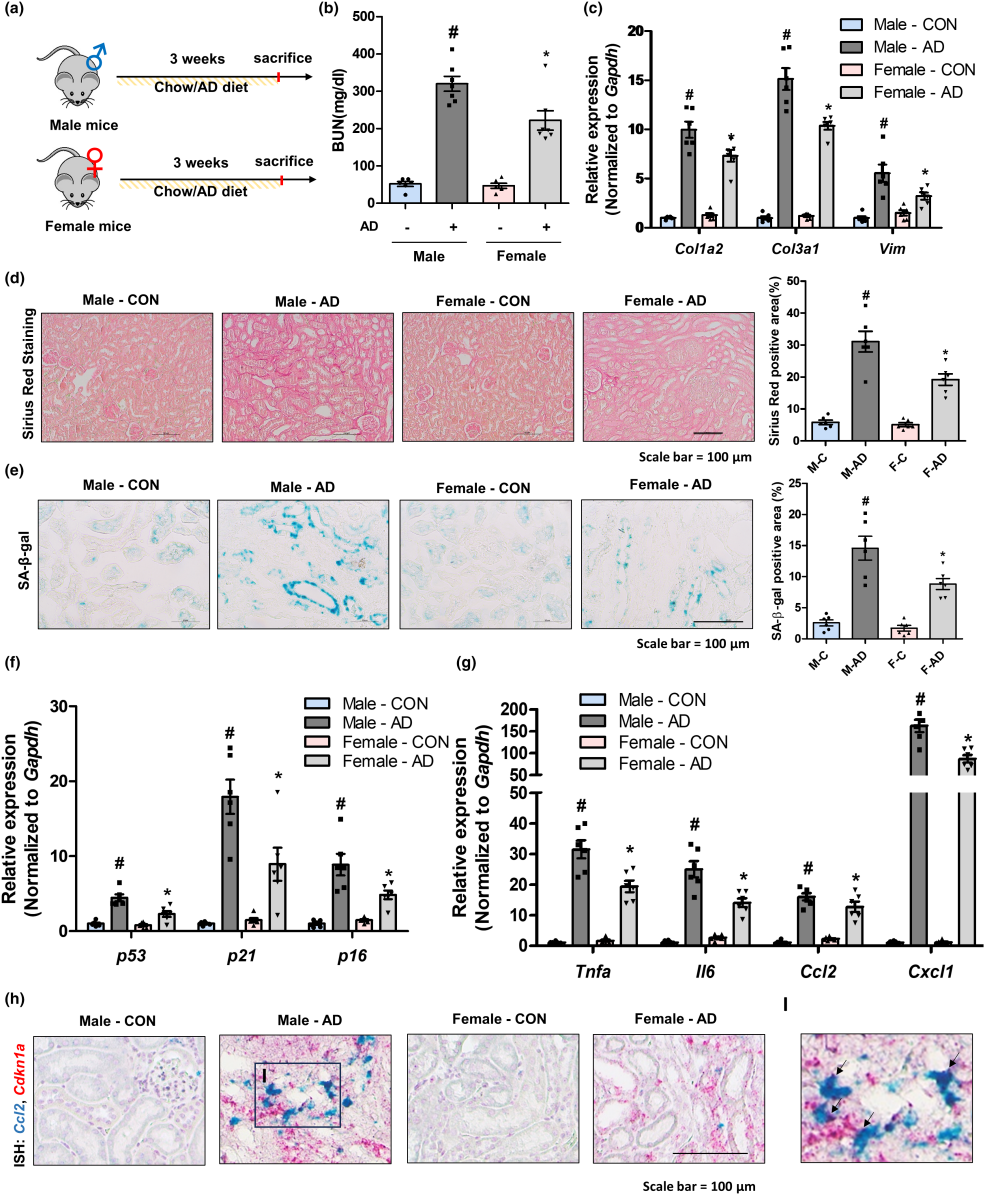
Fibrotic response is severe in male mouse kidneys and is associated with increased epithelial senescence. (a) Study design of 0.25% adenine diet (AD)‐fed kidney fibrosis‐inducing experiments in male and female mice. (b) Changes of BUN in four different groups. #*p* < 0.05 compared with male control group. *p < 0.05 compared with male AD‐fed group. (c) Relative mRNA expression of *Col1a2*, *Col3a1*, and *Vim*. #*p* < 0.05 compared with male control group. *p < 0.05 compared with male AD‐fed group. (d) Representative images of SR staining in the kidney sections of different groups. The area positive for SR staining were quantified for each group. #*p* < 0.05 versus male control group. *p < 0.05 versus male AD group. (e) Representative staining images show SA‐β‐gal activity in the kidney sections of different groups. The area positive for SA‐β‐gal staining were quantified for each group. #*p* < 0.05 versus male control group. *p < 0.05 versus male AD group. (f) Relative mRNA expressions of senescence markers including *p16*, *p21*, and *p53*. #*p* < 0.05 compared with male control group. *p < 0.05 compared with male AD‐fed group. (g) Relative mRNA expressions of SASP‐related genes (*Tnfa*, *Il6*, *Ccl2*, and *Cxcl1*). #*p* < 0.05 compared with male control group. *p < 0.05 compared with male AD‐fed group. (h) Representative dual‐ISH staining images of *Ccl2* (green) and *p21* (red) genes in different groups. (i) Magnified image of dual‐ISH staining from male AD‐treated mice. Arrows highlight cells positive for both *Ccl2* (green) and *Cdkn1a* (red).

To identify the role of cellular senescence in a general CKD model, we analyzed an adenine diet (AD)‐fed kidney fibrosis model in both male and female mice (Figure 2a). Serum BUN levels were significantly increased in both male and female mice‐fed AD, but the increase was higher in male mice (Figure 2b). The extent of fibrosis determined by gene expression and SR staining indicated severe damage to the kidneys of male mice fed the AD diet (Figure 2c,d). SA‐β‐gal‐positive signals were highly detectable in the tubule epithelial regions of AD‐fed mice kidney (Figure 2e). The levels of senescence‐related genes were significantly increased upon feeding with AD in both male and female kidneys, and higher levels were observed in male mouse kidneys (Figure 2f). The expression levels of inflammation‐related genes showed a similar trend in the kidneys of AD‐fed mice (Figure 2g). Similar to the aging model, senescent tubule cells expressing Cdkn1a also expressed Ccl2 (Figure 2h,i). Collectively, these data indicate that male mice are more susceptible to CKD injury and tubule epithelial senescence is also detectable, and contributes to inflammation in a CKD mouse model.

### 3.4 PAR2 expression is associated with cellular senescence and inflammation in diseased kidney

**FIGURE 3:**
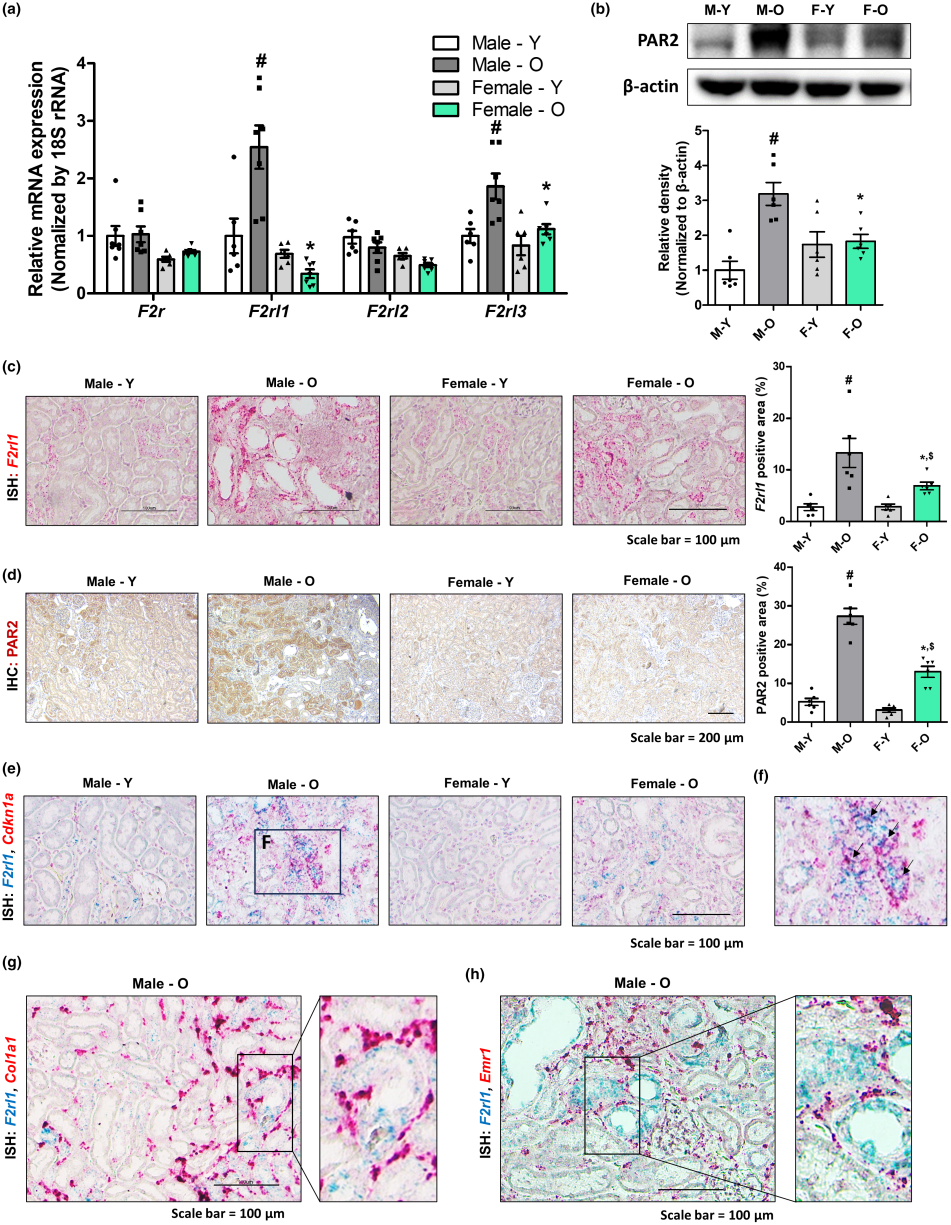
PAR2 expression is associated with cellular senescence and inflammation in fibrotic kidney. (a) Relative mRNA expression of *F2r*, *F2rl1*, *F2rl2*, and *F2rl3*. #*p* < 0.05 versus male young rats. *p < 0.05 versus male aged rats. (b) Renal protein expression levels of PAR2 were detected using western blotting in different groups. β‐Actin was used as internal control. Relative PAR2 expressions were quantified using densitometry. #*p* < 0.05 versus male young rats. *p < 0.05 versus male aged rats. (c) Representative ISH images of *F2rl1* (red) gene in the kidney sections of four groups. The area positive for ISH staining were quantified for each group. #*p* < 0.05 versus male young rats. *p < 0.05 versus male aged rats. $*p* < 0.05 versus female young rats. (d) Representative images of kidney sections immunostained with PAR2 antibody in different groups. The area positive for IHC staining were quantified for each group. #*p* < 0.05 versus male young rats. *p < 0.05 versus male aged rats. $*p* < 0.05 versus female young rats. (e) Representative dual‐ISH images of *F2rl1* (green) and *p21* (red) genes in different groups. (f) Magnified image of dual‐ISH staining from male aged rats. Arrows highlight cells positive for both *F2rl1* (green) and *Cdkn1a* (red). (g) Representative dual‐ISH staining images of *F2rl1* (green) and *Col1a1* (red) genes in male aged rat kidneys. (h) Representative dual‐ISH staining images of *F2rl1* (green) and *Emr1* (red) genes in male aged rat kidneys.

To investigate the relationship between PAR2 and tubular senescence, changes in the expression of the PAR family genes were examined in aged rat kidneys. The expression of the *F2rl1* gene increased the most in aged male rat kidneys compared with that in young male rat kidneys (Figure 3a). We further confirmed that PAR2 protein expression was increased in aged male kidneys (Figure 3b). Histologic analysis by ISH and immunohistochemistry further showed that PAR2 was mainly expressed in tubular epithelial cells (Figure 3c,d). In aged male kidneys, an increased *number of F2rl1*‐ and *Cdkn1a*‐positive cells were localized to the same tubular epithelial region, suggesting an association between tubular PAR2 expression and senescence (Figure 3e,f). In addition, *Col1a1*‐ or *Emr1*‐positive cells were present near the *F2rl1*‐positive tubular cells, suggesting a plausible role for PAR2 in kidney inflammation and fibrosis (Figure 3g,h).

We further confirmed that increased PAR2 expression is associated with cellular senescence in a CKD model. PAR2 gene and protein expression was significantly increased in the kidneys of AD‐fed mice (Figure S2A,B). PAR2 expression was mainly increased in tubular epithelial cells and was associated with cellular senescence and fibrosis (Figure S2C–E). Another cisplatin‐induced CKD model was analyzed to confirm the role of PAR2 in kidney senescence (Figure S3A). Cisplatin treatment significantly increased PAR2 gene and protein expression (Figure S3B,C). PAR2 expression was mainly increased in tubular epithelial cells and was associated with cellular senescence, inflammation, and fibrosis (Figure S3D,H). These data suggest that PAR2 expression is increased in aging kidneys and CKD models, and is associated with cellular senescence, inflammation, and fibrosis.

### 3.5 PAR2 activation induces cellular senescence and chemokine expression in renal tubule epithelial cells

**FIGURE 4:**
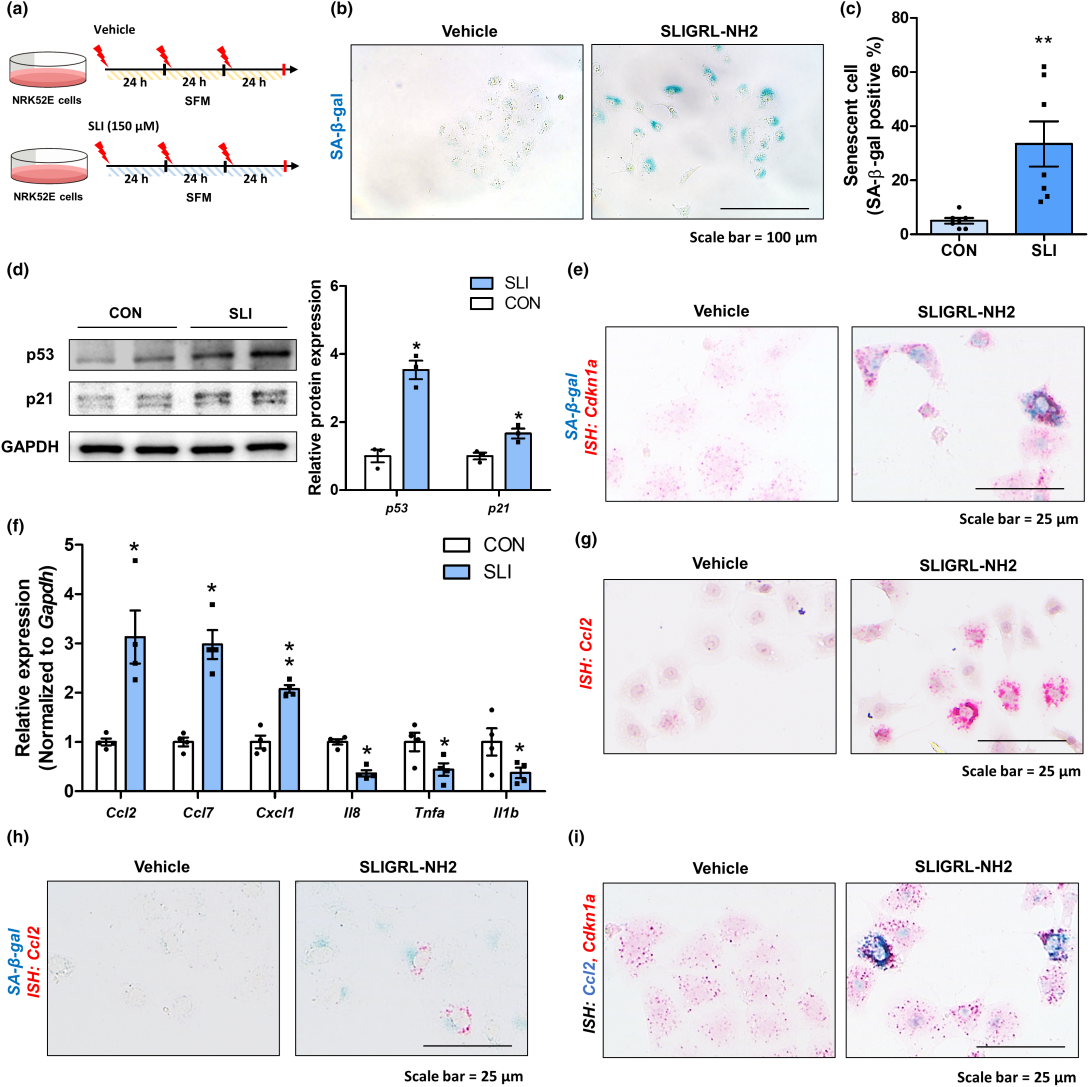
PAR2 activation promotes senescence and enhances chemokine expression in renal epithelial cells. (a) NRK52E renal epithelial cells were subjected to a triple treatment regimen with 150 μM SLIGRL‐NH2 (SLI), administered at 24‐h intervals, for a total duration of 72 h. Control cells were treated with vehicle for 72 h. (b) Representative images of SA‐β‐gal staining with or without PAR2 activation. (c) Quantification of SA‐β‐gal positive cells in cells with or without PAR2 activation. **p < 0.01 versus control group. (d) Western blots show protein levels of p53 and p21 with or without PAR2 activation. GAPDH was used as internal control. Relative protein expressions were quantified using densitometry. *p < 0.05 versus control group. (e) Representative images of double staining with *Cdkn1a* ISH staining and followed by SA‐β‐gal staining. (f) Relative mRNA expression of *Ccl2*, *Ccl7*, *Cxcl1*, *Il8*, *Tnfa*, and *Il1b* in NRK52E cells. *p < 0.01 versus control group. (g) Representative ISH images stained with *Ccl2* (red) probe in the cells. (h) Representative pictures of double staining with *Ccl2* ISH staining and followed SA‐β‐gal staining. (i) Representative dual ISH staining images of *Ccl2* (green) and *Cdkn1a* (red) gene in the cells.

We next performed in vitro experiments using NRK52E rat renal tubular epithelial cells. First, we examined whether PAR2 expression is increased in the cellular senescence model (Figure S4A). Cisplatin treatment significantly increased cellular senescence, and senescent cells showed increased PAR2 gene and protein levels, and Cdkn1a‐positive cells showed higher PAR2 expression in the senescent cells (Figure S4B–E). Next, we examined whether PAR2 activation induces cellular senescence in tubular epithelial cells. Treatment with the PAR2 agonist (SLIGRL‐NH2, SLI, 150 μM) at 24 h intervals with a total of three treatments resulted in a significant increase in senescence (Figure 4a–d). Using *Cdkn1a* ISH staining and followed by SA‐β gal staining, we confirmed that SA‐β‐gal‐positive senescent cells express p21 gene expression (Figure 4e). Interestingly, the gene expression of chemokines was significantly increased, whereas that of other cytokines was decreased (Figure 4f). The increased Ccl2 were further confirmed using ISH analysis, and SA‐β‐gal‐positive cells expressed Ccl2 (Figure 4g,h). When Ccl2 and Cdkn1a were detected simultaneously, Cdkn1a‐positive senescent cells highly expressed Ccl2 (Figure 4i). Collectively, PAR2 activation induces cellular senescence and chemokine expression in renal tubule epithelial cells.

### 3.6 PAR2‐mediated cellular senescence is associated with defective fatty acid oxidation

**FIGURE 5:**
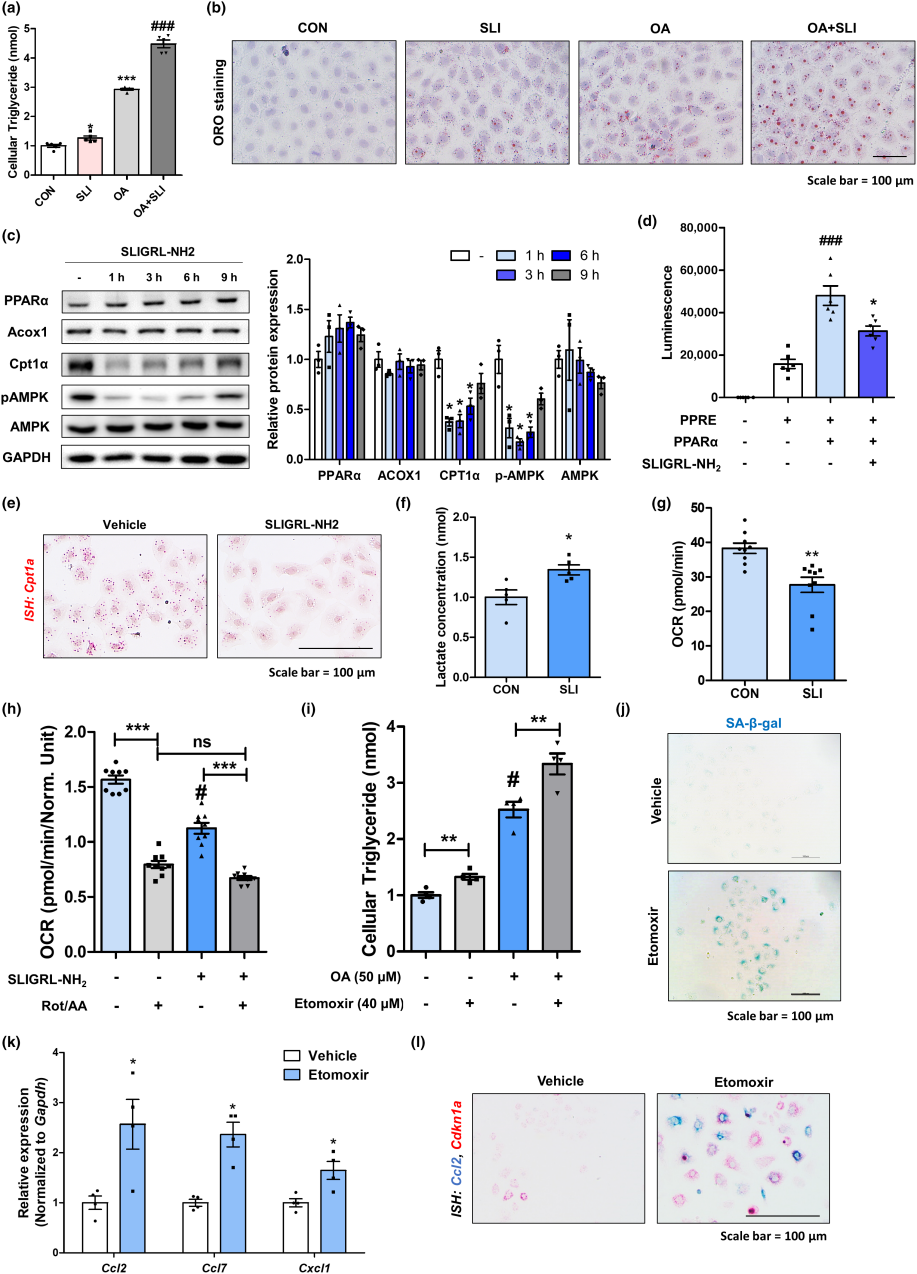
PAR2‐mediated cellular senescence is associated with defective fatty acid oxidation. (a) Cellular triglyceride contents were quantified in NRK52E cells treated with 150 μM of SLIGRL‐NH2 (SLI) or/and 50 μM of oleic acid (OA). *p < 0.05 and ***p < 0.001 versus control group. ###*p* < 0.001 versus OA‐treated group. (b) Lipid accumulation was visualized by Oil red O staining in NRK52E cells treated with SLI (150 μM) or/and OA (50 μM). (c) Protein levels of PPARα, Acox1, Cpt1α, phosphorylated AMPK, and AMPK were measured using western blotting in SLI‐treated cells. Relative protein expressions were quantified using densitometry. *p < 0.05 versus control group. (d) NRK52E cells were transfected with PPARα and PPRE plasmid for 24 h, followed by treatment with SLIGRL‐NH2. PPARα activity was measured using PPRE luciferase activity. ###*p* < 0.001 versus PPRE‐transfected group. *p < 0.05 versus PPRE + PPARα‐transfected group. (e) Representative ISH images stained with *Cpt1a* (red) probe in the cells. (f) The level of lactate in NRK52E cells was quantified with or without PAR2 activation. *p < 0.01 versus control group. (g) Cellular oxygen consumption rates (OCR) were measured using Seahorse systems under PAR2‐activated condition. **p < 0.01 versus control group. (h) OCR were measured using Seahorse systems under PAR2‐activated condition with or without Rotenone and Antimycin A treatment. #*p* < 0.05 versus control group. ***p < 0.001 between two groups. (i) Cellular triglyceride contents were quantified in NRK52E cells treated at designated conditions. **p < 0.01 between two groups. #*p* < 0.05 versus control group. (j) Representative images of SA‐β‐gal staining under etomoxir‐treated condition. (k) Relative mRNA expression of chemokines (*Ccl2, Ccl7*, and *Cxcl1*) in NRK52E cells treated with or without Etomoxir. *p < 0.01 versus vehicle group (l) *p < 0.01 versus vehicle group. Representative dual ISH images of *Ccl2* (green) and *Cdkn1a* (red) genes under etomoxir‐treated condition.

We hypothesized that PAR2 activation induces cellular senescence by decreasing FAO activity in renal tubular epithelial cells. In NRK52E cells, the activation of PAR2 significantly increased lipid accumulation, particularly in the presence of oleic acid treatment (Figure 5a,b). Interestingly, the protein levels of Cpt1α, one of the PPARα target proteins, decreased after PAR2 activation (Figure 5c). Furthermore, phosphorylated AMPK, an upstreaming signaling of PPARα, was also markedly decreased by a PAR2 activation (Figure 5c). Although PPARα protein levels were not altered by PAR2 activation, we found that PPARα transcriptional activity and *Cpt1a* gene expression levels were reduced following PAR2 activation (Figure 5c–e). Next, we quantitatively analyzed changes in metabolism via observing changes in glycolysis and OCR of the cells. The lactate concentration was higher after PAR2 activation than that in the control group, implying increased glycolysis (Figure 5f). In contrast, PAR2 activation significantly decreased OCR levels, indicating that the cells utilized anaerobic glycolysis instead of the FAO pathway to produce ATP under PAR2‐activated conditions (Figure 5g). In addition, inhibition of oxidative phosphorylation by rotenone/antimycin A treatment resulted in a lower OCR reduction under PAR2 activation, implying that PAR2 treatment reduced the oxygen consumption capacity of the cells (Figure 5h). To confirm whether the maintenance of FAO activity is critical for cellular senescence, we used etomoxir, a potent Cpt1α inhibitor. Etomoxir pretreatment significantly increased lipid accumulation, senescence, and chemokine expression in the cells (Figure 5i–l). Collectively, these results suggest that PAR2‐mediated cellular senescence is associated with defective FAO in renal tubular epithelial cells.

### 3.7 Defective FAO pathway is associated with PAR2 expression and cellular senescence in diseased kidney

We further investigated the relationship between defective FAO and senescence in diseased kidneys. Aged rat kidneys showed increased lipid accumulation in the tubular region with decreased Cpt1α and Acox1 levels (Figure S5A–C). Double immunofluorescence staining confirmed that PAR2 expression increased in the tubules of aged rat kidneys, and PAR2‐positive tubules had lower Cpt1α expression (Figure S5D). Senescent cells expressing *Cdkn1a* showed a lower expression of *Cpt1a*, while *Cdkn1a*‐negative cells showed a higher expression of *Cpt1a* (Figure S5E). These data indicate that a defective FAO pathway is associated with PAR2 expression and cellular senescence in aged kidneys.

Consistent with the aging model, decreased protein and mRNA expression levels of Cpt1α and Acox1 were detected in the AD‐fed kidneys (Figure S5F,G). PAR2 expression was high in the renal tubules of AD‐fed mice, and PAR2‐positive tubules showed lower protein expression of Cpt1α (Figure S5H). The co‐detection of *Cpt1a* and *Cdkn1a* showed a similar tendency in the aging model (Figure S5I). We also observed that *Col1a1*‐positive myofibroblasts were highly detectable near *Cpt1a‐*negative tubules, implying that fibrosis was severe near metabolically impaired tubules (Figure S5J). Collectively, these data suggest that a defective FAO pathway is associated with PAR2 expression and cellular senescence in both aging and diseased kidneys.

### 3.8 PAR2 deficiency alleviates FAO and protects from senescence in CKD model

To confirm the detrimental role of PAR2 in CKD, we utilized PAR2 KO mice model. Wild‐type (WT) and PAR2 KO mice were fed 0.25% AD for 3 weeks. Compared to WT mice, PAR2 KO mice showed less senescence‐associated gene and protein expression, less SA‐β‐gal activity in the kidneys (Figure S6A–C). PAR2 KO mice showed less decrease in PPARα, CPT1α, and Acox1 gene and protein level, and less increase in lipid accumulation compared to WT mice kidney under AD‐fed condition (Figure S6D–F). Dual ISH staining results showed that PAR2 KO mice had a higher number of *Cpt1a*‐positive tubules, whereas fewer tubule cells were positive for *Cdkn1a* expression (Figure S6G). These data suggest that PAR2 deficiency significantly alleviates defects in the FAO pathway and cellular senescence in the tubular epithelial region of fibrotic kidneys.

PAR2 KO mice showed a lower expression of kidney damage‐related genes in the kidneys (Figure S7A). The expression of chemokine genes in the kidneys of PAR2 KO mice exhibited less increase, which was accompanied by decreased expression of macrophage marker genes in the kidneys (Figure S7B,C). Dual ISH analysis indicated that a decrease in senescent cells in the tubules of PAR2 KO mouse kidneys correlated with reduced expression of *Ccl2* and decreased macrophage infiltration (Figure S7D,E). PAR2 KO mice also showed less fibrosis in the kidney (Figure S7F–H). Similar to the AD‐fed model, PAR2 KO mice were protected from cisplatin‐induced kidney damage, senescence, inflammation, and fibrosis (Figures S8 and S9). Collectively, these data suggest that mice lacking PAR2 are shielded from CKD progression owing to a reduction in cellular senescence.

### 3.9 PAR2 deficiency ameliorates cellular senescence, fibrosis, and inflammation in aged mice kidneys

**FIGURE 6:**
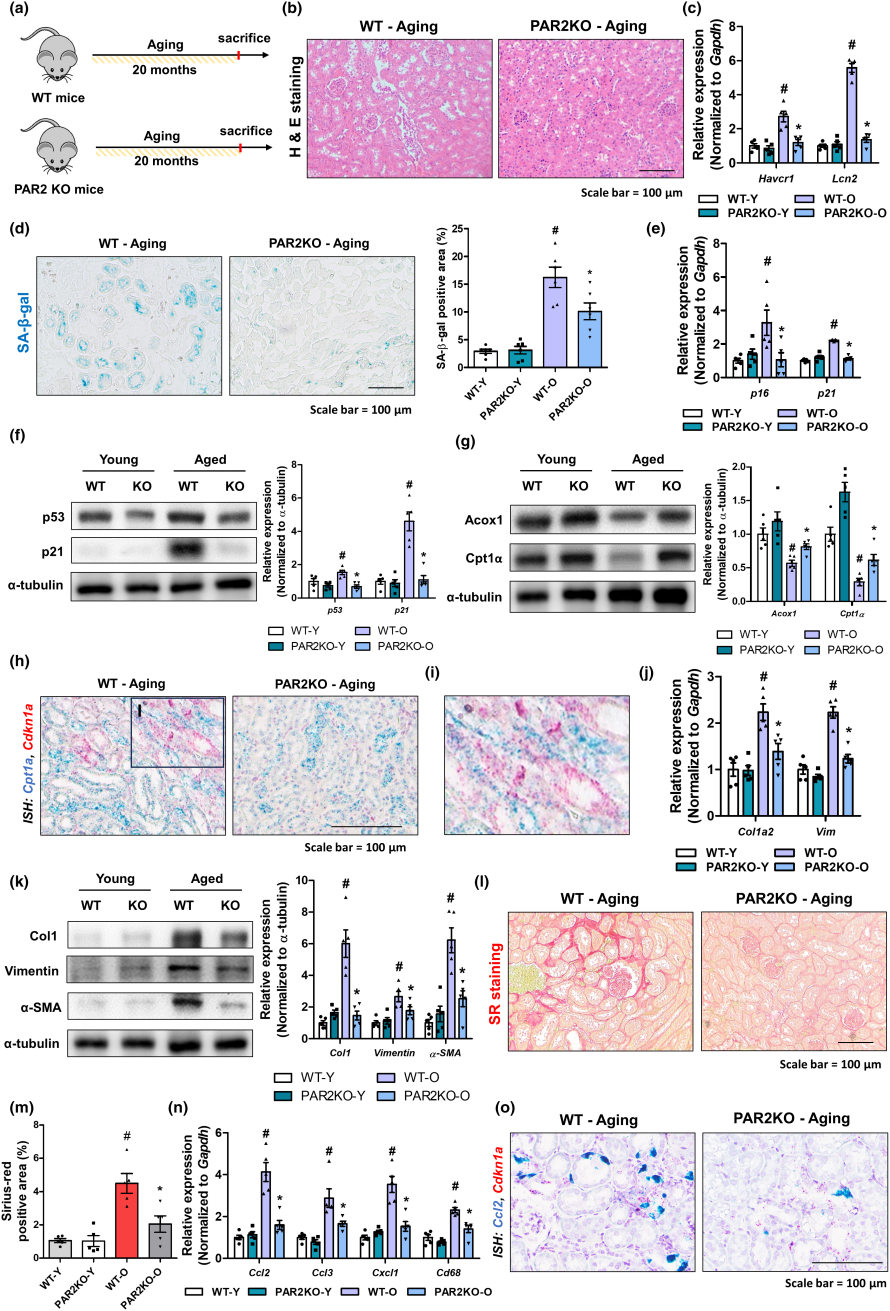
PAR2 deficiency alleviates age‐related cellular senescence and fibrosis in mice. (a) Experimental scheme for PAR2 KO aging experiments. (b) Representative H&E staining images of mouse kidney samples from different groups. (c) Relative mRNA expression of *Havcr1 (Hepatitis A virus cellular receptor1)* and *Lcn2 (Lipocalin 2)*. #*p* < 0.05 compared with young WT group. *p < 0.05 compared with aged WT group. (d) Representative images showing SA‐β‐gal activity in the kidney sections. The area positive for SA‐β‐gal staining were quantified for each group. #*p* < 0.05 versus WT young mice. *p < 0.05 versus WT aged mice. (e) Relative mRNA expression of *p16* and *p21*. #*p* < 0.05 compared with young WT group. *p < 0.05 compared with aged WT group. (f) The protein expression of p21 and p53 were detected using western blotting in the kidneys of different groups. α‐tubulin was used as internal control. Relative protein expressions were quantified using densitometry. #*p* < 0.05 versus WT young mice. *p < 0.05 versus WT aged mice. (g) Renal protein expression of ACOX1 and CPT1α were detected using western blotting in different groups. α‐tubulin was used as internal control. Relative protein expressions were quantified using densitometry. #*p* < 0.05 versus WT young mice. *p < 0.05 versus WT aged mice. (h) Representative dual‐ISH images of *Cpt1a* (green) and *Cdkn1a* (red) genes in two groups. (i) Magnified image of dual‐ISH staining from WT aged mice. (j) Relative mRNA expression of *Col1a2* and *Vim*. #*p* < 0.05 compared with young WT group. *p < 0.05 compared with aged WT group. (k) Protein levels of Col1, Vimentin, and α‐SMA were detected using western blotting in the kidneys. α‐tubulin was used as internal control. Relative protein expressions were quantified using densitometry. #*p* < 0.05 versus WT young mice. *p < 0.05 versus WT aged mice. (l) Representative images of SR staining of kidney sections. (m) Quantification of fibrosis extent detected by Sirius red staining using ImageJ. #*p* < 0.05 compared with young WT group. *p < 0.05 compared with aged WT group. (n) Relative mRNA expression of *Ccl2, Ccl3, Cxcl1*, and *Cd68*. #*p* < 0.05 compared with young WT group. *p < 0.05 compared with aged WT group. (o) Representative image of dual ISH staining images of *Ccl2* (green) and *Cdkn1a* (red) genes in two groups.

To investigate the role of PAR2 in kidney aging, we established an aging mouse model using PAR2 KO and WT mice (Figure 6a). In the PAR2 KO mice, aging‐induced increased tubule dilation, epithelial cell shedding in the kidneys, and increased expression of genes were significantly attenuated, indicating less kidney damage than in aged mice (Figure 6b,c). The extent of renal cellular senescence was less increased in PAR2 KO kidney compared to WT kidney (Figure 6d–f). Aged kidneys showed a significant decrease in Cpt1α and Acox1 protein levels, whereas PAR2 KO kidneys showed a smaller decrease (Figure 6g). We further found that senescent epithelial cells expressing *Cdkn1a* showed lower *Cpt1a* expression in aged kidneys (Figure 6h,i). PAR2 KO kidneys showed less fibrosis than WT kidneys (Figure 6j–m). Finally, we observed that PAR2 KO kidneys showed lower inflammatory gene expression than WT kidneys (Figure 6n). In WT‐aged kidneys, senescent tubule cells showed *Ccl2* expression, whereas PAR2 KO kidneys were less senescent, *Ccl2* expressing cells (Figure 6o). Collectively, these data indicate that PAR2 activation during aging increases cellular senescence, inflammation, and fibrosis in the kidneys.

## 4 DISCUSSION

Cellular senescence is a common feature of aging and age‐related diseases that contributes to tissue dysfunction in different organs. Senescent cells can exert detrimental effects through multiple mechanisms (Chung, Dhillon, et al., 2019; Chung, Kim, et al., 2019; Lopez‐Otin et al., 2023). They can disrupt tissue function, impair tissue repair and regeneration, promote chronic inflammation, and alter the local microenvironment (Di Micco et al., 2021). In this study, we demonstrated that PAR2 activation directly induces cellular senescence in renal tubule epithelial cells. Senescent cells exhibit highly inflammatory phenotypes and secrete major chemokines. Using in vivo aging and kidney injury models, we found that PAR2 deficiency significantly protected the kidneys from cellular senescence, inflammation, and fibrosis. Understanding the role of PAR2 in cellular senescence may provide new insights for developing therapeutic strategies targeting aging and kidney disease.

Cellular senescence in kidneys has been well reported since its first observation. Melk et al. reported that senescence in rat kidneys increases with age and is associated with structural and functional changes (Melk et al., 2003). They found increased expression of p16^INK4a^ protein and SA‐β‐gal activity in aged tubule epithelial regions. Subsequently, cellular senescence has been linked to pathologic changes during the development of CKD. Data from animal models have suggested that senescent tubular epithelial cells contribute to ischemia–reperfusion injury, septic shock‐induced AKI, diabetic kidney disease (DKD), and IgA nephropathy (Chen, Qiu, et al., 2021; Chen, Zhang, et al., 2021; Kim, Kim, et al., 2021; Kim, Puranik, et al., 2021; Liu et al., 2012, 2015). Recently, mechanisms underlying CKD‐induced epithelial senescence have been elucidated. Tubular epithelial cell senescence can be induced by overexpression of Wnt9a and activation of the Wnt‐β‐catenin pathway, which promotes epithelial‐to‐mesenchymal transition and consequent fibrosis (Gong et al., 2021; Luo et al., 2018). Decreased FFAR4 expression has been detected in AKI, and activation of FFAR4 by TUG891 alleviates the senescence of tubular epithelial cells via the AMPK/SIRT3 signaling pathway (Yang et al., 2022). The inhibition of tubular senescence using genetic or pharmacologic methods significantly reduces kidney damage, inflammation, and fibrosis in various kidney disease models (Li et al., 2018, 2023). Furthermore, senolytic treatment restored the regenerative phenotype of the kidneys, suggesting that senescent cells represent a potential target to protect the kidneys (Docherty et al., 2020).

Renal tubule cells exhibit high baseline energy consumption to perform their functions efficiently, relying predominantly on FAO as their primary energy source (Kang et al., 2015; Rinaldi et al., 2022). The importance of the FAO process is particularly emphasized in pathologic conditions. Kang et al. reported that impaired FAO pathways in tubular cells are associated with the development of renal interstitial fibrosis (Kang et al., 2015). The authors discovered that reduced FAO causes lipid deposition with ATP depletion, leading to fibrosis development. The importance of the FAO process was further confirmed using an aging model (Chung et al., 2018). More recently, Miguel et al., demonstrated that renal tubule CPT1α overexpression protects from kidney fibrosis by restoring FAO pathway and mitochondrial homeostasis (Miguel et al., 2021). In contrast, the genetic ablation of CPT1α aggravates tubular injury and interstitial fibrosis in animal models (Yuan et al., 2021). In this study, we found that defective FAO was involved in the PAR2 signaling pathway. In tubular epithelial cells, PAR2 activation significantly decreased FAO via PPARα‐dependent CPT1α downregulation. These observations led us to investigate whether PAR2‐mediated metabolic changes are associated with senescence in epithelial cells.

An expanding body of literature suggests a close association between cellular senescence and altered metabolic state (Wiley & Campisi, 2021). Senescent cells exhibit multiple modifications in their metabolic state, including altered fatty acid metabolism. In an oncogene‐induced senescence model, upregulated β‐oxidation was necessary for the secretion of SASP (Quijano et al., 2012). During replicative senescence in myoblasts, the levels of several acyl‐carnitines were found to be increased, suggesting that senescent cells rely heavily on β‐oxidation (Baraibar et al., 2016). Therapy‐induced cellular senescence in normal and tumor cells showed an increase in β‐oxidation regulators at both the proteomic and transcriptional levels (Flor et al., 2017). These data suggest that fatty acids are important substrates for energy production in senescent cells. On the other hand, there are several evidences that inhibition of β‐oxidation induces cellular senescence. Loss of CPT1C in lung fibroblasts promotes lipid accumulation and induces senescence via lipotoxicity (Chen, Qiu, et al., 2021; Chen, Zhang, et al., 2021). The deficiency of CPT1 also aggravates cardiomyocyte senescence and pressure overload‐induced cardiac hypertrophy owing to increased lipotoxicity (He et al., 2012). A plausible explanation is that lung fibroblasts and cardiomyocytes rely predominantly on lipid oxidation; when this process is inhibited, it leads to lipotoxicity and cellular senescence. Our observations also support the notion that the inhibition of CPT1 and β‐oxidation is critical for cellular senescence. Activation of PAR2 significantly alters cellular lipid metabolism, causing a decrease in CPT1α expression, followed by a reduced OCR. Critically, CPT1 inhibition was sufficient to induce cellular senescence. Because renal tubular epithelial cells are highly dependent on lipid substrates are prone to become senescent when β‐oxidation is inhibited.

The impact of gender disparities on CKD progression has been substantiated through both clinical observations and experimental investigations. Studies have revealed a lifetime risk of ESRD up to 50% higher in men compared to women across various racial and ethnic groups (Albertus et al., 2016). Moreover, recent prospective studies examining sex differences in CKD progression have shown that women exhibit a lower risk of both CKD progression and mortality compared to men (Ricardo et al., 2019). In numerous experimental models of kidney disease, male animals have displayed accelerated progression (Lima‐Posada et al., 2017). Additionally, evidence suggests that age‐related kidney changes are more prominent in male rodents (Erdely et al., 2003). Our own findings corroborate these results. In both aging and adenine diet‐induced kidney injury models, male rodents exhibited structural alterations and declines in kidney function. Furthermore, hormonal manipulations in experimental kidney disease models have replicated the gender‐related effects on disease progression (Carrero et al., 2018). This suggests that female sex hormones may decelerate CKD progression, while male hormones may exacerbate it. Given our discovery of higher PAR2 expression in both aging and adenine diet‐induced kidney injury models, and considering estrogen's known ability to mitigate inflammatory responses, further studies could explore whether estrogen could suppress PAR2 and PAR2‐related cellular senescence.

In conclusion, our study identified PAR2 as an important regulator of renal tubule epithelial senescence, which amplifies inflammation and fibrosis during aging and CKD development (Figure S10). Elevated PAR2 expression was observed in the renal tubule epithelial cells of both aged and CKD animal models, which correlated with tubule cell senescence. Under in vitro conditions, PAR2 activation induces cellular senescence and increases chemokine expression in renal tubule cells. Further mechanistic studies revealed that PAR2‐mediated cellular senescence is associated with defective fatty acid oxidation. Using PAR2 KO mouse models, we discovered that the absence of PAR2 provides kidney protection in both CKD and aging models. Collectively, these findings provide new perspectives on the mechanisms underlying renal senescence, and suggest that directing interventions against PAR2 could be a promising therapeutic strategy for the management of kidney injury.

## AUTHOR CONTRIBUTIONS

**Conceptualization:** Hae Young Chung and Ki Wung Chung. **Data curation**: Sugyeong Ha, Hyun Woo Kim, and Kyung Mok Kim. **Investigation:** Sugyeong Ha, Hyun Woo Kim, Byeong Moo Kim, Jeongwon Kim, Minjung Son, and Doyeon Kim. **Resources:** Jaewon Lee, Young‐Suk Jung, Hak Sun Yu, Hae Young Chung, and Ki Wung Chung. **Supervision**: Hae Young Chung and Ki Wung Chung. **Writing**: Sugyeong Ha and Ki Wung Chung.

## FUNDING INFORMATION

This study was supported by the National Research Foundation of Korea (NRF) grants funded by the Korean government (MSIT) (No. RS‐2023‐00208093, No. 2023R1A2C2006035, and NRF‐2021R1I1A1A0105215311).

## CONFLICT OF INTEREST STATEMENT

The authors have declared that no conflict of interest exists.

## Supporting information


Appendix S1.

## Data Availability

All data used in this study are available in this article.
