# Supplementary material for: PAR2‐mediated cellular senescence promotes inflammation and fibrosis in aging and chronic kidney disease
Source: Aging Cell. 2024 Apr 30;23(8):e14184. doi: 10.1111/acel.14184 (PMC11320361; doi:10.1111/acel.14184)
Supplement: Supplementary file 1 — Appendix S1. [file ACEL-23-e14184-s001.zip › acel14184-sup-0001-Supplementary Figures.pdf]

Supplementary Figures

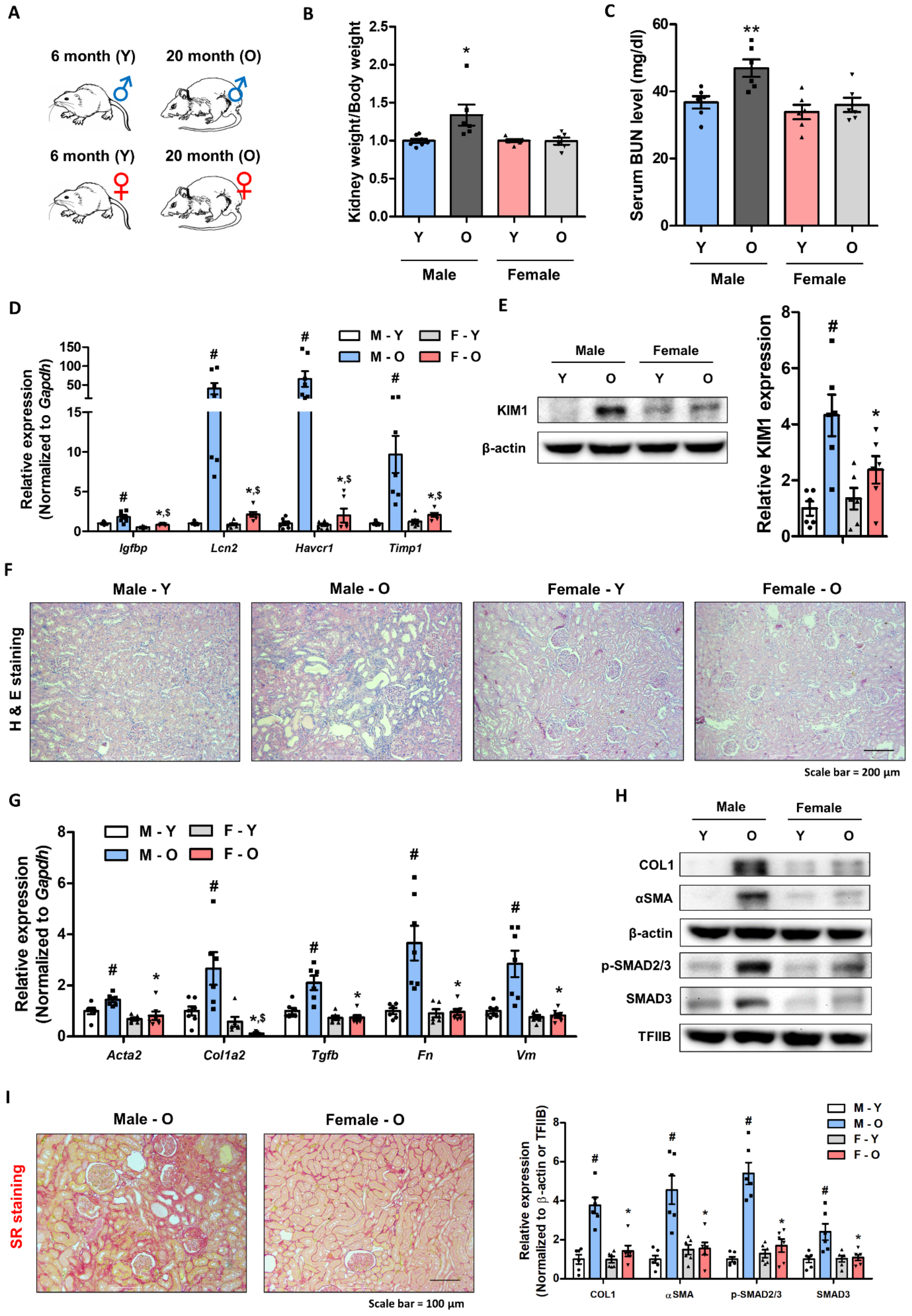

Supplementary Figure 1. Differences in age-related renal changes in male and female SD rats. (A) Study design of the aging experiments. (B) Kidney weight/body weight ratio of SD rats. *$p < 0.05$ versus male young rats. (C) Contents of blood urea nitrogen (BUN) in serum. *$p < 0.05$ versus male young rats. (D) Relative mRNA levels of *Igfbp*, *Lcn2*, *Havcr1*, and *Timp1* in the kidneys of male and female SD rat aged 6 and 20 months. #$p < 0.05$ versus male young rats. *$p < 0.05$ versus male aged rats. $\$p < 0.05$ versus female young rats. (E) Representative western blots showing the renal expression of KIM1 in four groups. β-actin was used as internal control. Relative protein expressions were quantified using densitometry. #$p < 0.05$ versus male young rats. *$p < 0.05$ versus male aged rats. (F) Representative images of rat kidney samples from different groups with H&E staining. (G) Relative mRNA expression levels of fibrosis-related genes including *Acta2*, *Col1a2*, *Tgfb*, *Fn*, and *Vm*. #$p < 0.05$ versus male young rats. *$p < 0.05$ versus male aged rats. $\$p < 0.05$ versus female young rats. (H) COL1 and α-SMA was detected in the cytosol, and p-SMAD2/3 and SMAD3 was detected in the nucleus using western blotting. β-actin, and TFIIB was used as internal control. Relative protein expressions were quantified using densitometry. #$p < 0.05$ versus male young rats. *$p < 0.05$ versus male aged rats. (I) Representative images of kidney samples stained with Sirius Red staining.

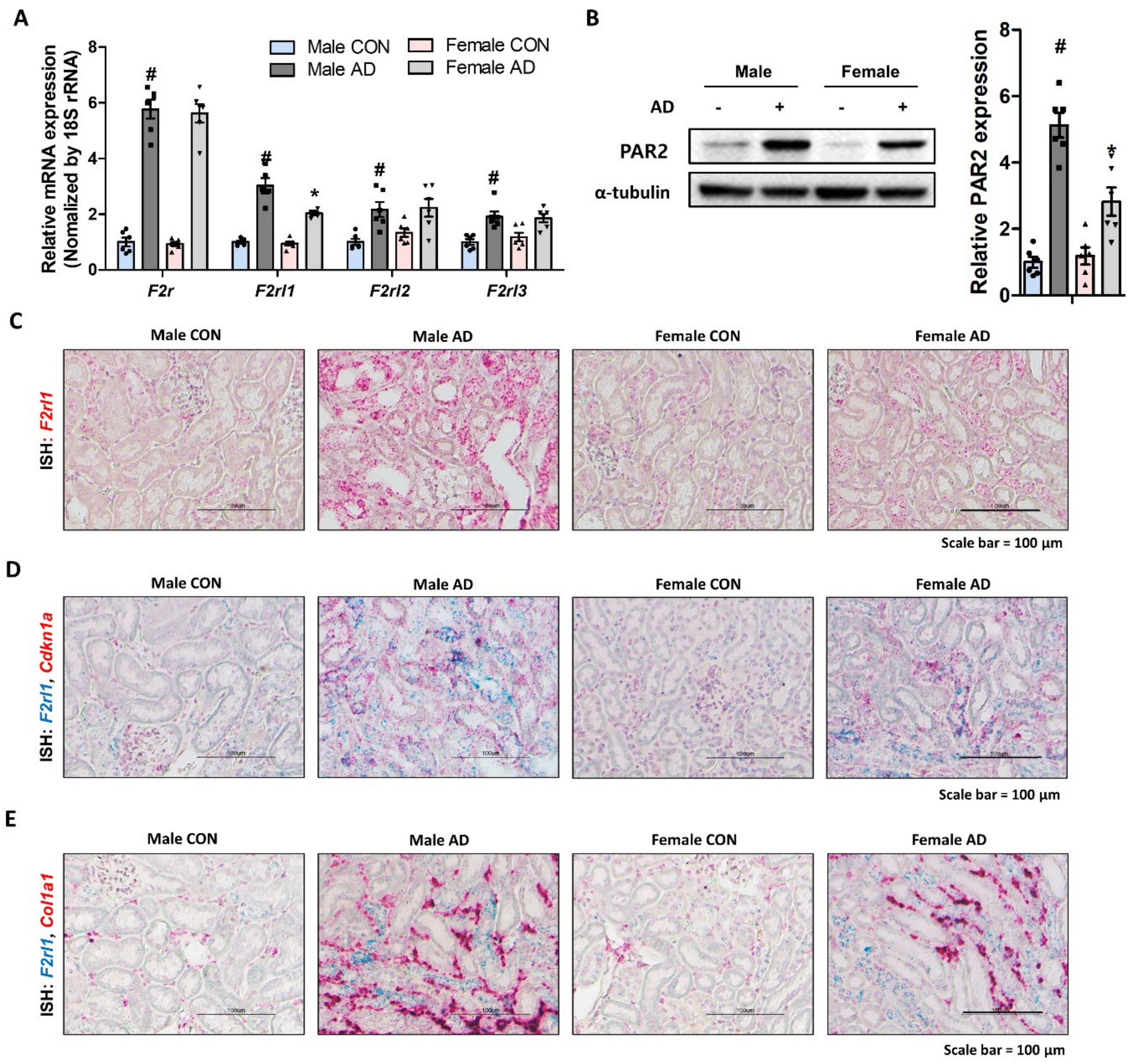

Supplementary Figure 2. PAR2 expression is associated with cellular senescence in fibrotic kidney. (A) Relative mRNA expression of *F2r*, *F2rl1*, *F2rl2* and *F2rl3*. #p < 0.05 compared with male control group. *p < 0.05 compared with male AD-fed group. (B) Representative western blots show protein levels of PAR2. α-tubulin was used as internal control. Relative protein expressions were quantified using densitometry. #p < 0.05 versus male control group. *p < 0.05 versus male AD-fed group. (C) Representative images of ISH staining with *F2rl1* (red) probe in the kidneys. (D) Representative dual ISH images of *F2rl1* (green) and *Cdkn1a* (red) genes in the kidneys. (E) Representative dual ISH staining images of *F2rl1* (green) and *Col1a1* (red) genes in the kidneys.

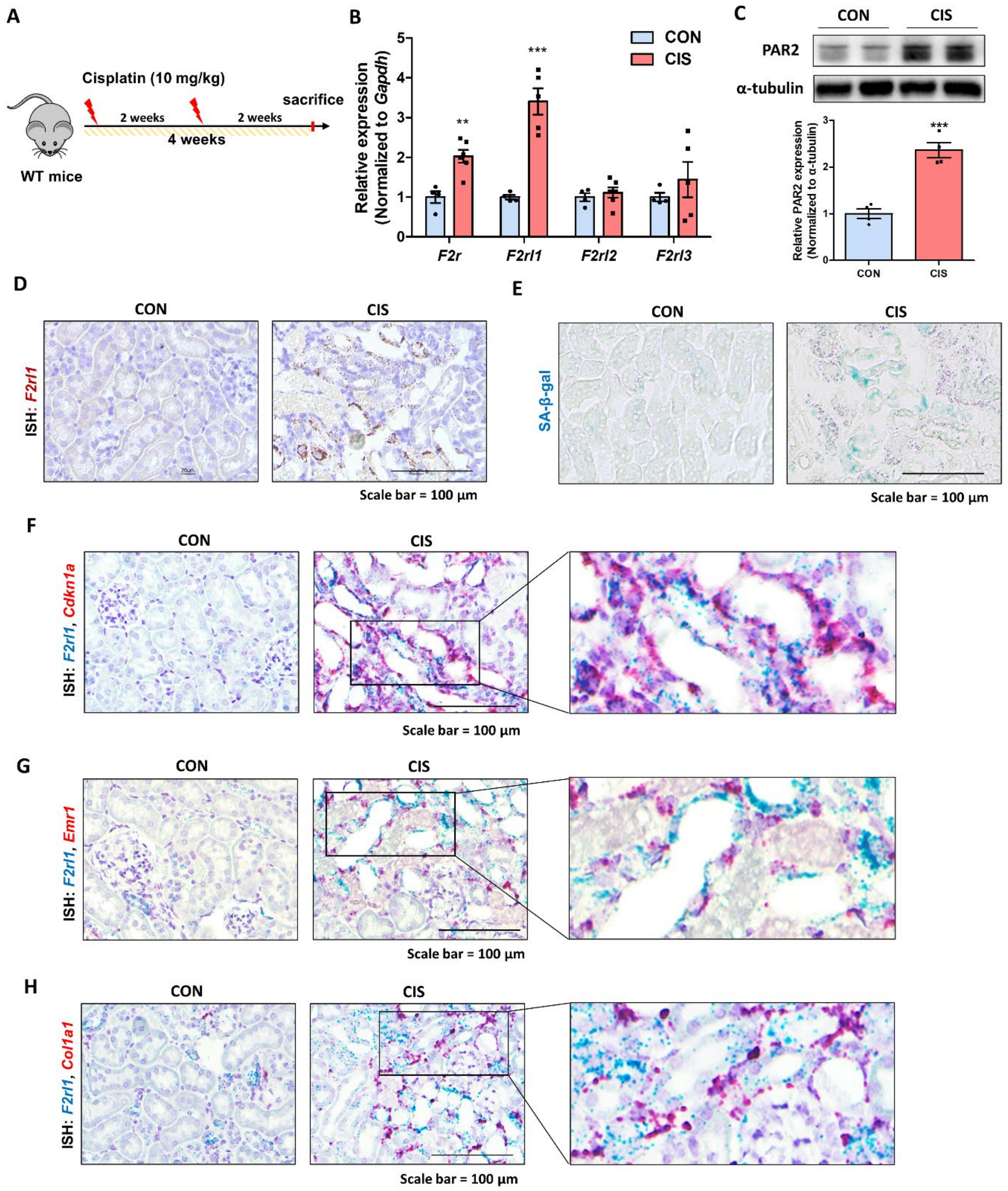

Supplementary Figure 3. Cisplatin induces PAR2 activation and triggers senescence in renal tubules. (A) Study design of cisplatin experiments. (B) Relative mRNA expression of *F2r*, *F2rl1*, *F2rl2*, and *F2rl3*. $**p < 0.01$ and $***p < 0.001$ versus control group. (C) Representative western blots showing the renal expression of PAR2 in two groups. α-tubulin was used as internal control. Relative protein expressions were quantified using densitometry.

***$p < 0.001$ versus control group. (D) Representative ISH images stained with *F2rl1* (brown) probe in the kidneys of two groups. (E) Representative images of renal SA-β-gal activity in two groups. (F) Representative dual-ISH images of *Ccl2* (green) and *Cdkn1a* (red) genes in different groups. (G) Representative dual-ISH images of *F2rl1* (green) and *Emr1* (red) genes in two groups. (H) Representative dual-ISH images of *F2rl1* (green) and *Col1a1* (red) genes in different groups.

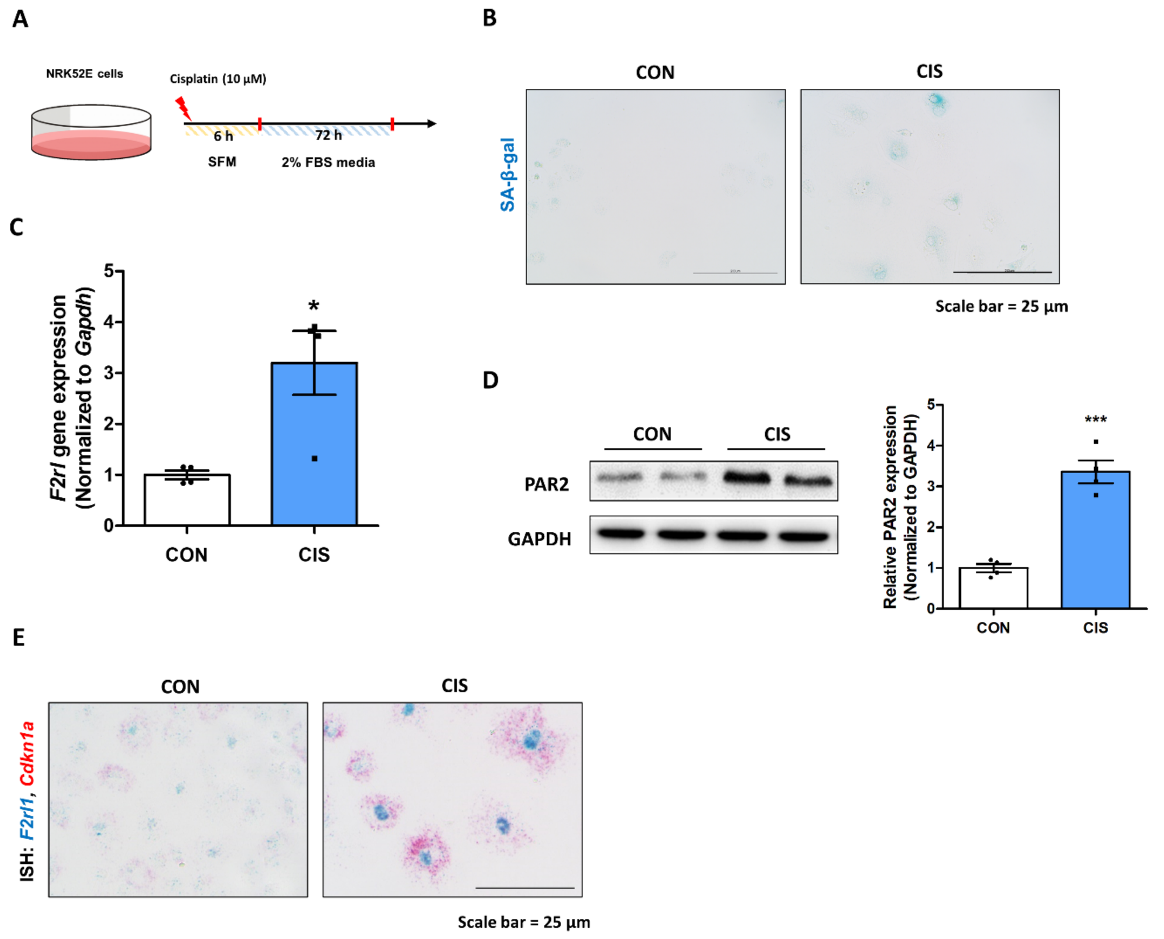

Supplementary Figure 4. Cisplatin treatment leads to NRK52E cell senescence, and senescent cells show elevated expression of PAR2. (A) NRK52E renal epithelial cells were treated with cisplatin (10 μM) for 6 h, and the medium was replaced with 2% FBS media and incubated for 72 h. (B) SA-β-gal staining assay was performed in NRK52E cells. (C) qRT-PCR analysis was performed to determine the mRNA levels of *F2rl1*. *$p < 0.05$ compared with control group. (D) Expressions of PAR2 protein were measured using western blotting in NRK52E cells. GAPDH was used as loading control. Relative protein expressions were quantified using densitometry. ***$p < 0.001$ versus control group. (E) Representative ISH staining images showing the expression levels of *F2rl1* (green) and *Cdkn1a* (red) genes in NRK52E cells.

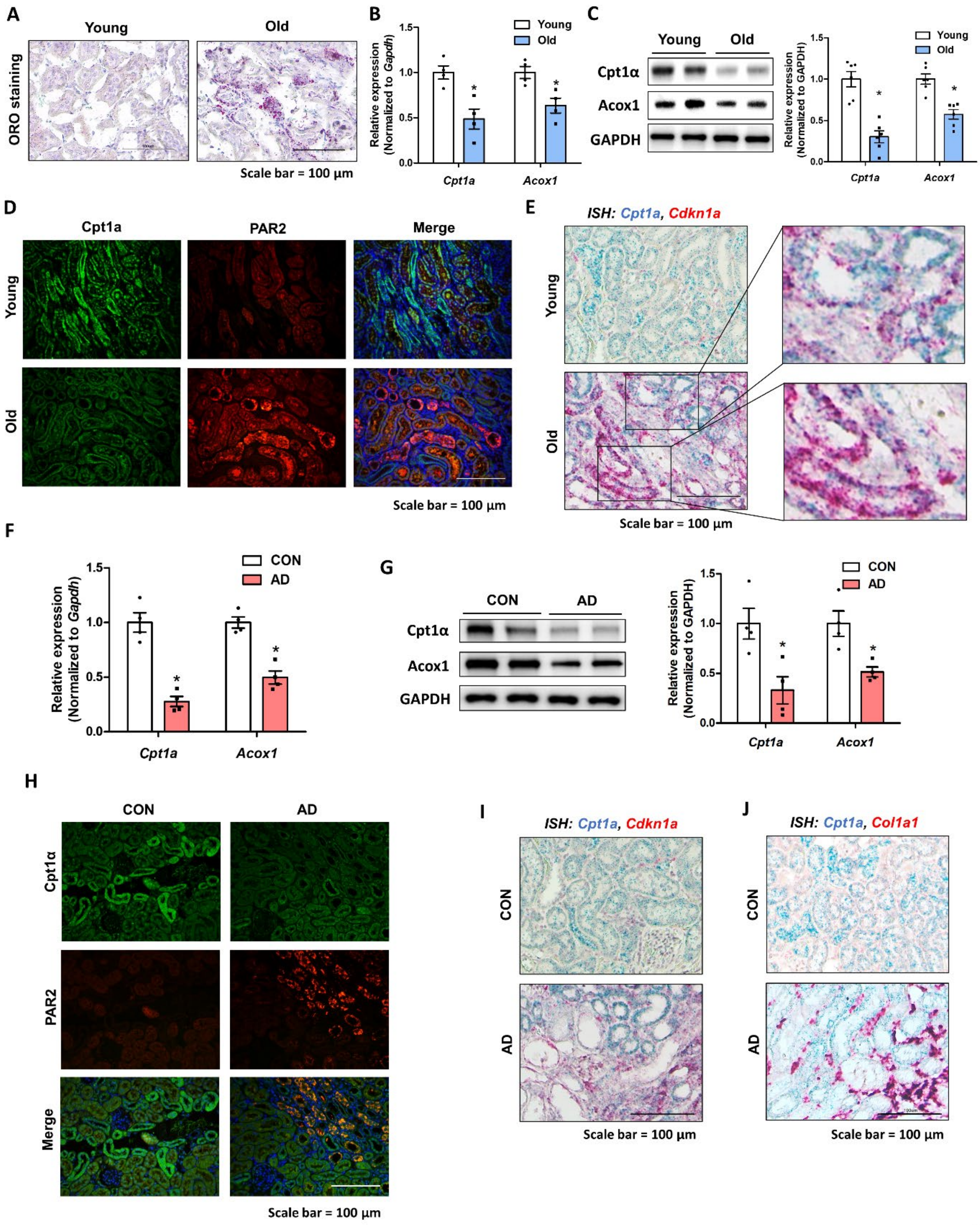

Supplementary Figure 5. Renal senescence is associated with defective fatty acid oxidation in fibrotic kidney. (A) Representative ORO-staining images in kidney sections from young and aged rat. (B) Relative mRNA levels of FAO-related genes (*Cpt1a* and *Acox1*) in young and aged rat kidney. *p < 0.05 versus young rats. (C) Western blot measurement of

Cpt1α and ACOX1 expression in young and aged rat kidney. GAPDH was used as internal control. Relative protein expressions were quantified using densitometry. *$p < 0.05$ versus young group. (D) Representative immunofluorescence images of CPT1α and PAR2 in young and aged kidneys. Scale bar, 100 μm. (E) Representative double-ISH staining images of *Cpt1a* (green) and *p21* (red) genes in the kidney of young or aged rat. (F) Relative mRNA expression of *Cpt1a* and *Acox1* in control and AD-induced fibrotic kidney. *$p < 0.05$ versus control group. (G) Renal protein expression of CPT1α and ACOX1 were detected using western blotting in different groups. GAPDH was used as internal control. Relative protein expressions were quantified using densitometry. *$p < 0.05$ versus control group. (H) Representative immunofluorescence staining images of the renal CPT1α and PAR2 expression in different groups. (I) Representative dual ISH images of *Cpt1a* (green) and *p21* (red) genes in different groups. (J) Representative dual ISH images of *Cpt1a* (green) and *Col1a1* (red) genes in different groups.

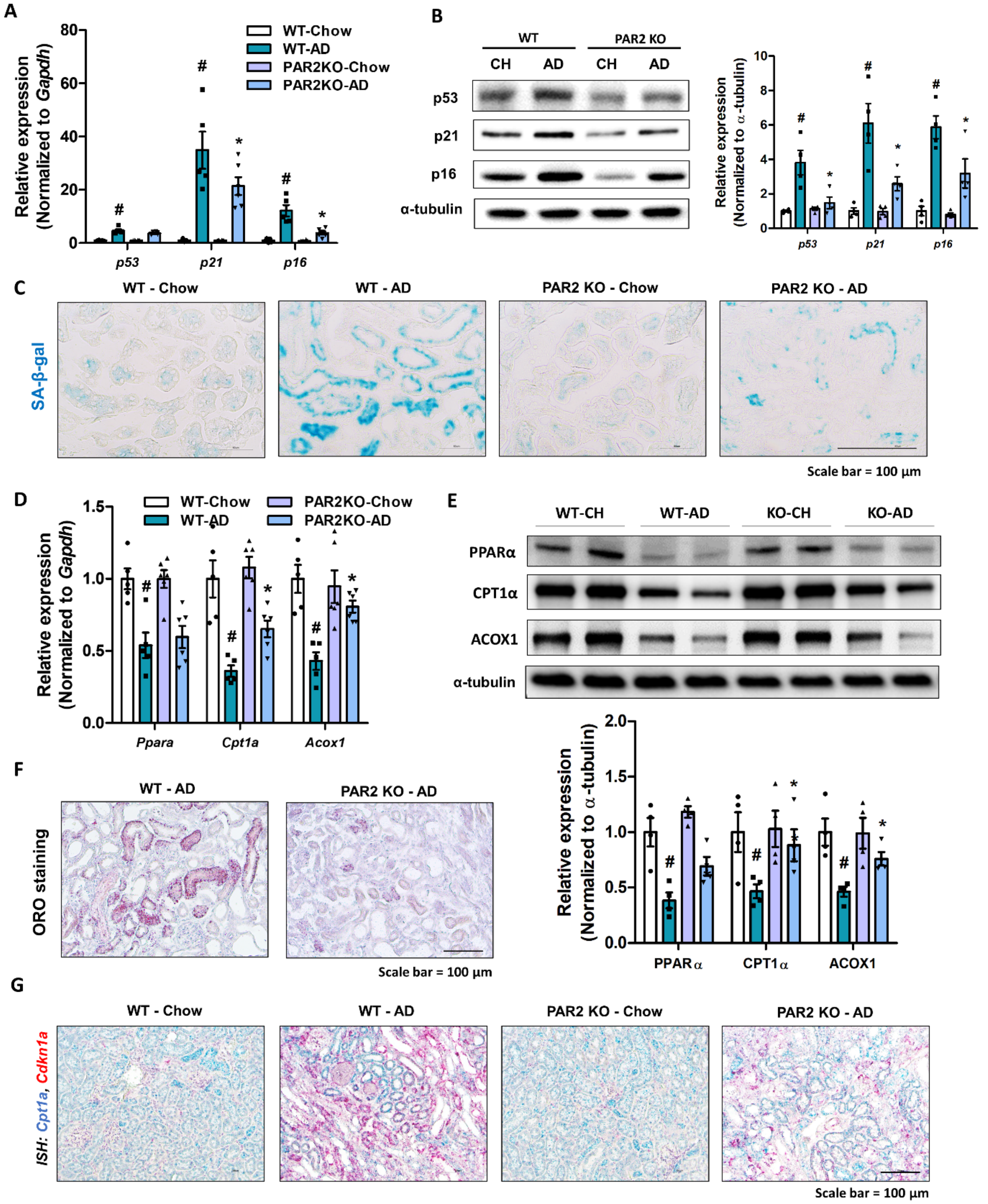

Supplementary Figure 6. PAR2 deficiency alleviates FAO and reduces senescence in adenine diet-induced kidney disease model. (A) Relative mRNA expression of *p16*, *p21*, and *p53*. #p < 0.05 compared with chow diet-fed WT group. *p < 0.05 compared with AD-fed WT group. (B) Representative western blots showing the renal protein expression of p53, p21, and

p16 in different groups. α-tubulin was used as internal control. Relative protein expressions were quantified using densitometry. #p < 0.05 compared with chow diet-fed WT group. *p < 0.05 compared with AD-fed WT group. (C) Representative images showing SA-β-gal activity in different groups. (D) Relative mRNA expression of *Ppara, Cpt1a,* and *Acox1*. #p < 0.05 compared with chow diet-fed WT group. *p < 0.05 compared with AD-fed WT group. (E) Protein expression of renal CPT1α, ACOX1, and PPARα were detected using western blotting in different groups. α-tubulin was used as internal control. Relative protein expressions were quantified using densitometry. #p < 0.05 compared with chow diet-fed WT group. *p < 0.05 compared with AD-fed WT group. (F) The kidney sections were stained with ORO to visualize lipid accumulation. (G) Representative dual ISH staining images of *Cpt1a* (green) and *p21* (red) genes in different groups.

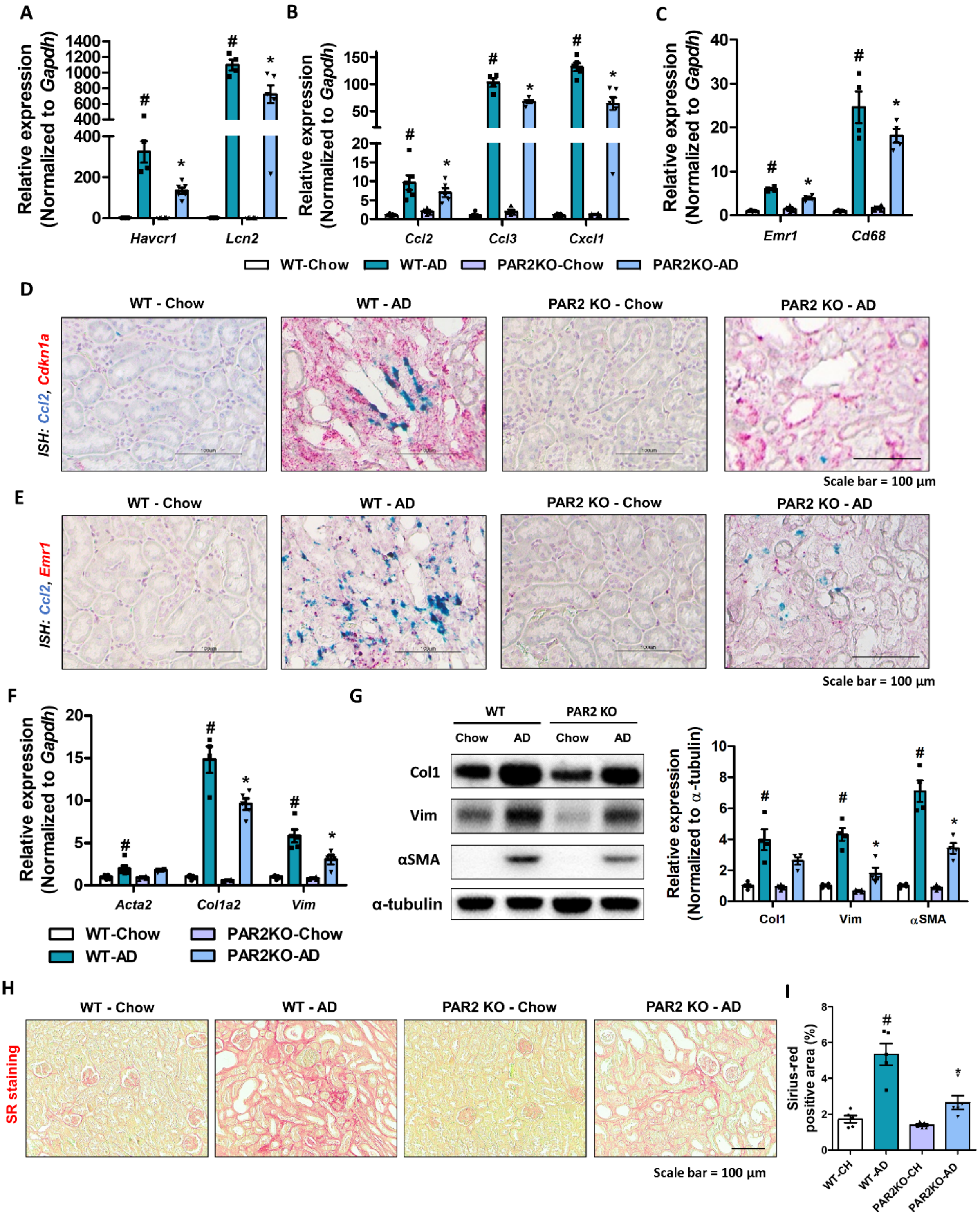

Supplementary Figure 7. PAR2 deficiency alleviates renal inflammation and fibrosis. (A) Relative mRNA expression of *Havcr1* and *Lcn2*. #p < 0.05 compared with chow diet-fed WT group. *p < 0.05 compared with AD-fed WT group. (B) Relative mRNA expression of *Ccl2*, *Ccl3*, and *Cxcl1*. #p < 0.05 compared with chow diet-fed WT group. *p < 0.05 compared with

AD-fed WT group. (C) Relative mRNA expression of *Emr1* and *Cd68*. #p < 0.05 compared with chow diet-fed WT group. *p < 0.05 compared with AD-fed WT group. (D) Representative dual-ISH images of *Ccl2* (green) and *p21* (red) genes in different groups. Scale bar, 100 µm. (E) Representative dual-ISH images of *Ccl2* (green) and *Emr1* (red) genes in different groups. (F) Relative mRNA expression of *Acta2*, *Col1a2*, and *Vim*. #p < 0.05 compared with chow diet-fed WT group. *p < 0.05 compared with AD-fed WT group. (G) Protein levels of Col1, VIM, and α-SMA were detected using western blotting in the kidneys. α-tubulin was used as internal control. Relative protein expressions were quantified using densitometry. #p < 0.05 compared with chow diet-fed WT group. *p < 0.05 compared with AD-fed WT group. (H) Representative images of SR staining of kidney sections from WT or PAR2KO mice. (I) Quantification of fibrosis extent calculated by SR staining-positive region using Image J. #p < 0.05 compared with chow diet-fed WT group. *p < 0.05 compared with AD-fed WT group.

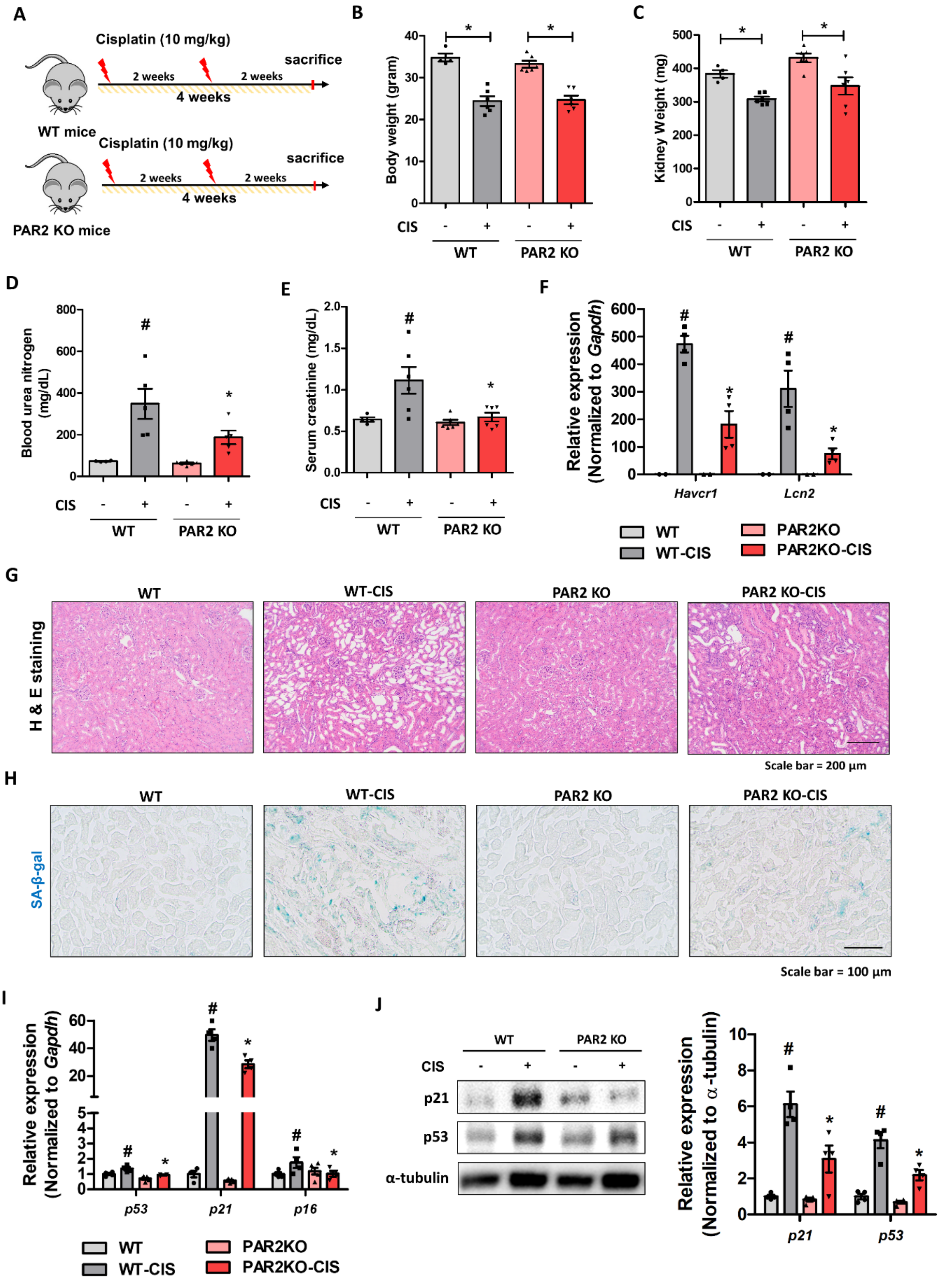

Supplementary Figure 8. PAR2 deficiency protects from cisplatin-induced kidney senescence. (A) Experimental design describing cisplatin-induced kidney injury in wildtype

and PAR2 KO mice. (B) Body weight. *p < 0.05 compared between two groups. (C) Kidney weight. *p < 0.05 compared between two groups. (D) Changes in serum BUN levels. #p < 0.05 versus WT control group. *p < 0.05 versus cisplatin treated WT group. (E) Changes of serum creatinine levels. #p < 0.05 versus WT control group. *p < 0.05 versus cisplatin treated WT group. (F) The mRNA expression levels of *Havcr1* and *Lcn2* were investigated using qRT-PCR. #p < 0.05 versus WT control group. *p < 0.05 versus cisplatin treated WT group. (G) Representative images of mouse kidney sections from WT and PAR2KO mice stained with H&E staining. (H) Representative images showing SA-β-gal activity in different groups. (I) Relative mRNA expressions of *p53*, *p21*, and *p16*. #p < 0.05 versus WT control group. *p < 0.05 versus cisplatin treated WT group. (J) Representative immunoblotting images of p21 and p53 in kidney tissues. Relative protein expressions were quantified using densitometry. #p < 0.05 versus WT control group. *p < 0.05 versus cisplatin treated WT group.

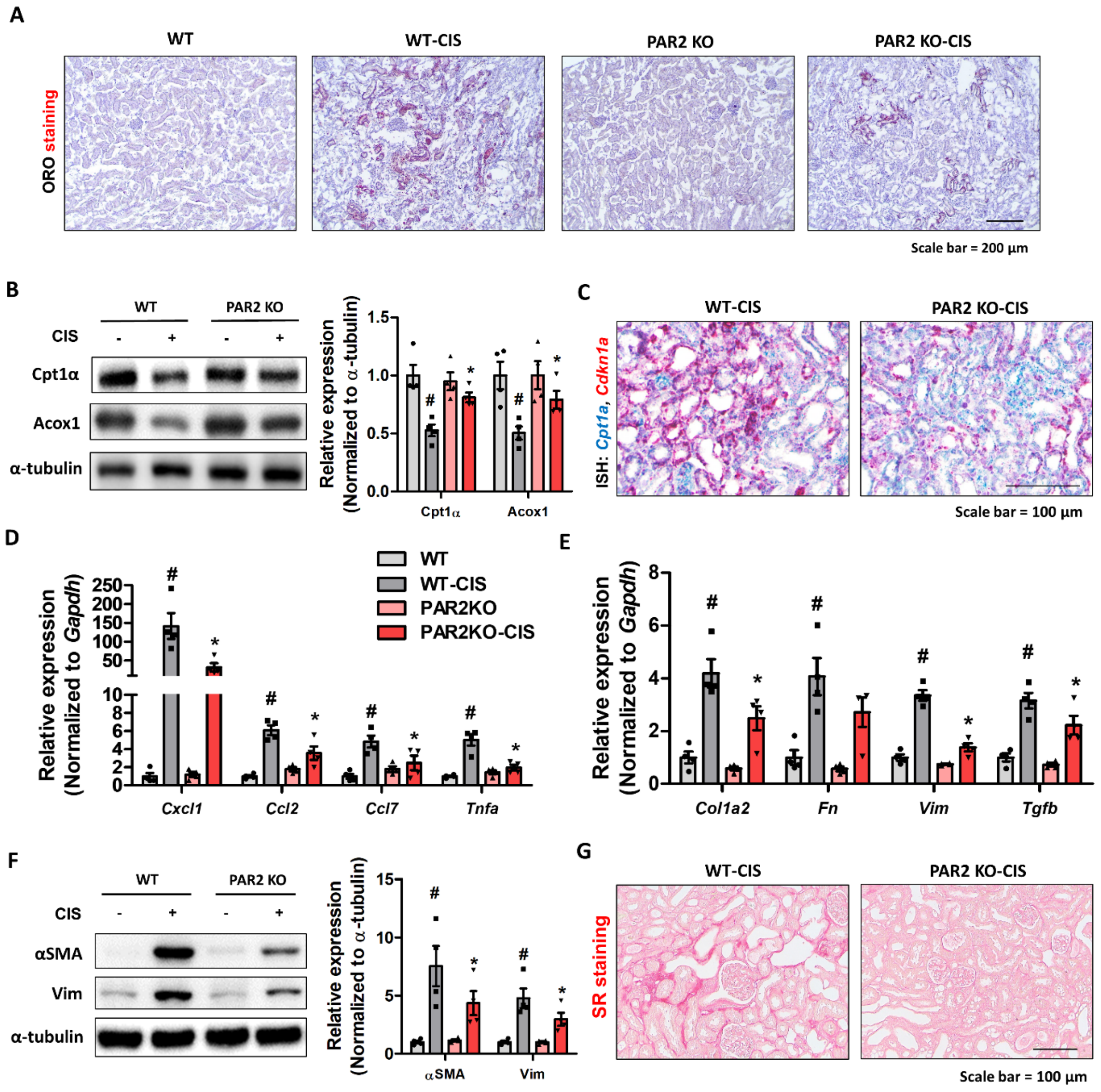

Supplementary Figure 9. PAR2 deficiency alleviates FAO and reduces fibrosis in cisplatin-induced kidney disease model. (A) Representative images of ORO staining of kidney samples from WT and PAR2KO mice. (B) Representative western blots showing the renal expression of Cpt1α and Acox1 in different groups. Relative protein expressions were quantified using densitometry. #p < 0.05 versus WT control group. *p < 0.05 versus cisplatin treated WT group. (C) Representative ISH image of mouse kidney sections from WT and PAR2KO mice stained with *Cpt1a* (green) and *Cdkn1a* (red) probe. (D) The mRNA expression levels of *Cxcl1*, *Ccl2*, *Ccl7*, and *Tnfa* were investigated using qRT-PCR. #p < 0.05 versus WT

control group. *p < 0.05 versus cisplatin treated WT group. (E) The mRNA expression levels of *Col1a2*, *Fn*, *Vim*, and *Tgfb* were investigated using qRT-PCR. #p < 0.05 versus WT control group. *p < 0.05 versus cisplatin treated WT group. (F) Representative western blots showing the renal expression of αSMA and Vimentin. α-tubulin was used as internal control. Relative protein expressions were quantified using densitometry. #p < 0.05 versus WT control group. *p < 0.05 versus cisplatin treated WT group. (G) Representative images of SR staining of kidneys from WT and PAR2KO mice.

Supplementary Tables

Supplementary Table 1. Information of primary antibodies used in Western blotting

Antibody	Company	Catalog number
KIM-1	Abcam	ab 47635
β-actin	Santa Cruz	sc 69879
COL1	Santa Cruz	sc 80760
α-SMA	Santa Cruz	sc 32251
p-Smad2/3	Santa Cruz	sc 101801
Smad3	Santa Cruz	sc 101154
TFIIB	Santa Cruz	sc 225
p53	Santa Cruz	sc 125
p21	Santa Cruz	sc 271610
p16	Abcam	ab 211542
GAPDH	Santa Cruz	sc 25778
PAR2	Santa Cruz	sc 13504
PPARα	Abcam	ab 8934
Acox1	Abcam	ab 184032
Cpt1α	Abcam	ab 128568
p-AMPK	Cell Signaling	#2535
AMPK	Santa Cruz	sc 74461
α-tubulin	Santa Cruz	sc 8035
Vimentin	Cell Signaling	#5741

Supplementary table 2. Primer sequences for qPCR

Mouse

Gene	*Forward (5'-3')*	*Reverse (3'-5')*
Col1a2	*CAGCTCCAGGAAGACCTCGA*	*GTAACAAGGGTGAGCCTGGC*
Col3a1	*ATCAAACACGCAAGGCCATG*	*AAGCAAACAGGGCCAATGTC*
Vim	*CAAGCCTGACCTCACTGCTG*	*CACCTGTCTCCGGTACTCGT*
p53	*AGAGACCGCCGTACAGAAGA*	*CTGTAGCATGGGCATCCTTT*
p21	*CGGTGGAACTTTGACTTCGT*	*CAGGGCAGAGGAAGTACTGG*
p16	*TCAACTACGGTGCAGATTCG*	*TCGCACGATGTCTTGATGTC*
Tnfa	*CGTCAGCCGATTTGCTATCT*	*CGGACTCCGCAAAGTCTAAG*
Il6	*AGTTGCCTTCTTGGGACTGA*	*TCCACGATTTCCCAGAGAAC*
Ccl2	*CCAGCAAGATGATCCCAATG*	*CTTCTTGGGGTCAGCACAGA*
Cxcl1	*GCTGGGATTCACCTCAAGAA*	*TGGGGACACCTTTTAGCATC*
Cpt1a	*GTGCTCTGAGGCCTTTGTCA*	*GGTCCAGGTAGAGCTCAGGC*
Acox1	*TTACCCAGCCCTGGCTTAAT*	*AGGTCACAGCTGTCCAACCA*
Ppara	*ATGCCAGTACTGCCGTTTTC*	*GGCCTTGACCTTGTTCATGT*
Havcr1	*CCGTGTCTCTGCTCACTACAG*	*CCAACTTTTAATTCCTATCCCTCT*
Lcn2	*CCAGTTCGCCATGGTATTTT*	*GGTGGGGACAGAGAAGATGA*
Ccl3	*ATGAAGGTCTCCACCACTGC*	*CCCAGGTCTCTTTGGAGTCA*
Emr1	*TCTGGGGAGCTTACGATGGA*	*GAATCCCGCAATGATGGCAC*
Cd68	*GGGGCTCTTGGGAACTACAC*	*GTACCGTCACAACCTCCCTG*
Acta2	*AGCTGTCTTTTTGGCCCATT*	*GGTTCTGGGCTCTGTAAGGC*
F2r	*CTCCTCAAGGAGCAGACCAC*	*AGACCGTGGAAACGATCAAC*
F2rl1	*GGACGCAACAACAGTAAAGGA*	*CAGAGAGGAGGTCAGCCAAG*
F2rl2	*GTGTACCAGCCAACATCGTG*	*CTCGCCAAATACCCAGTTGT*
F2rl3	*GCAGACCTTCCGATTAGCTG*	*CAGTCTGAGTGCATGGCTGT*
Ccl7	*AATGCATCCACATGCTGCTA*	*ATAGCCTCCTCGACCCACTT*
Fn	*CAACAACCGGAATTACACCG*	*GTCTCGGAGCTGGGAGTAGG*
Tgfb	*CCTCACCTCCATGTACCAGAA*	*TGGAAATGACCTTGTCAATGAG*
Gapdh	*TGCTGGTGCTGAGTATGTCG*	*AGTTGGTGGTGCAGGATGC*
18S rRNA	*GTGGTCTTGGTGTGCTGACC*	*GACAACAAGCTGCGTGAGGA*

Rat

Gene	*Forward (5'-3')*	*Reverse (3'-5')*
Igfbp	*CCATGGTGGTCTGGCAATACA*	*AAGCTGACAGGCAGGCAGAAT*
Lcn2	*GGTGGGAACAGAGAAAACGA*	*CAAGTGGCCGACACTGACTA*
Havcr1	*CGCGGTTCTATTTTGTTGGT*	*CAAAGCTCAGAGAGCCCATC*
Timp1	*GGTGGGGACAGAGAAGATGA*	*TCCCCAGAAATCATCGAGAC*
Acta2	*TTGTCCACCGCAAATGCTTC*	*AAGGCGCTGATCCACAAAAC*
Col1a2	*CGTCGTGCCTAGCAACATGC*	*AGTTCCCAGTAAGACCAGGG*
Tgfb	*AGAAGAACTGCTGTGTACGG*	*AAGGACCTTGCTGTACTGTG*
Fn	*ATTCCAATGGTGCCTTGTGC*	*TGCCGCACCATTTCATGTTG*
Vm	*CGCCATCAACACCGAGTTCA*	*CTCGGCCAGCAGGATTTTGT*
p53	*TCTCCCCAGCAAAAGAAAAA*	*CTTCGGGTAGCTGGAGTGAG*
p21	*AGCAAAGTATGCCGTCGTCT*	*ACACGCTCCCAGACGTAGTT*
p16	*AACGTCAAAGTGGCAGCTCT*	*CCCAGCGGAGGAGAGTAGAT*
Tnfa	*ATTGCTCTGTGAGGCGACTG*	*GGGGCTCTGAGGAGTAGACG*
Il1b	*AAAATGCCTCGTGCTGTCTG*	*CCACAGGGATTTTGTCGTTG*
Il6	*TCTCTCCGCAAGAGACTTCCA*	*ATACTGGTCTGTTGTGGGTGG*
Ccl2	*GCCAACTCTCACTG AGCCA*	*GCATCTGGCTGAGACAGCAC*
Ccl3	*TGCCCTTGCTGTTCTTCTCT*	*AAAGGCTGCTGGTCTCAAAA*
Ccl7	*ACAGCTGCTGCTTTCACCGT*	*TCAACCCACTTCTGATGGGCT*
F2r	*CTCCTCAAGGAGCAGACCAC*	*AGACCGTGGAAACGATCAAC*
F2rl1	*GGACGCAACAACAGTAAAGGA*	*CAGAGAGGAGGTCAGCCAAG*
F2rl2	*GTGTACCAGCCAACATCGTG*	*CTCGCCAAATACCCAGTTGT*
F2rl3	*GCAGACCTTCCGATTAGCTG*	*CAGTCTGAGTGCATGGCTGT*
Cxcl1	*AGACAGTGGCAGGGATTCAC*	*GGGGACACCCTTTAGCATCT*
Il8	*GAAGATAGATTGCACCGA*	*CATAGCCTCTCACACATTTC*
Cpt1a	*ATGACGGCTATGGTGTCTCC*	*GTGAGGCCAAACAAGGTGAT*
Acox1	*TTGGAAACCACTGCCACATA*	*CCCGTAGCACTCTCCTTGAG*
Gapdh	*TGGGGAAAAGCCTTGAAGAT*	*CACCGACCTTCACCATTTTG*
18S rRNA	*AGTCGGCATCGTTTATGGTC*	*CGCGGTTCTATTTTGTTGGT*
