# Supplementary figures and images for: PAR2‐mediated cellular senescence promotes inflammation and fibrosis in aging and chronic kidney disease
Source: Aging Cell. 2024 Apr 30;23(8):e14184. doi: 10.1111/acel.14184 (PMC11320361; doi:10.1111/acel.14184)

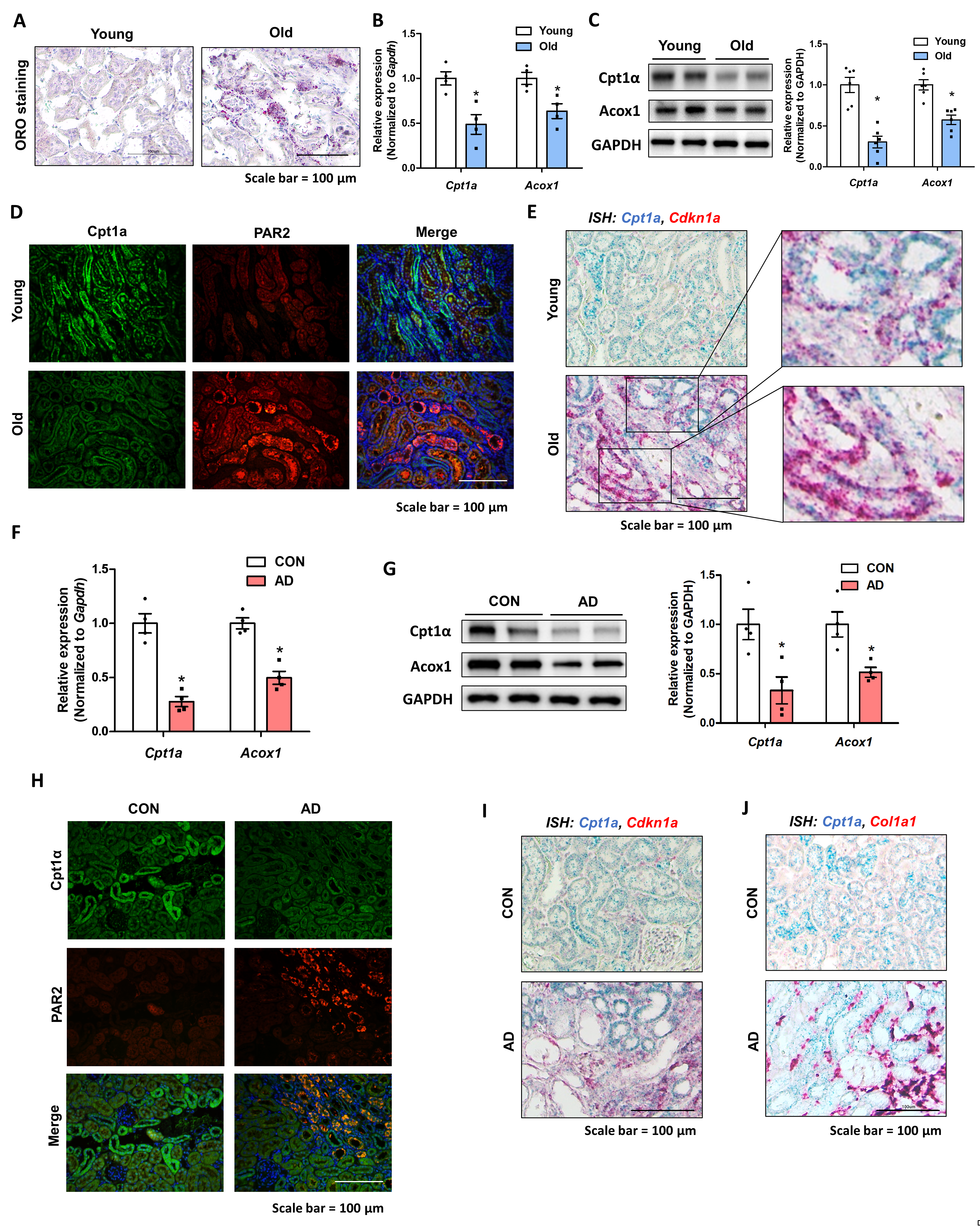

Supplement: Supplementary file 1 — Appendix S1. [file ACEL-23-e14184-s001.zip › S Figure 5 new.tif]

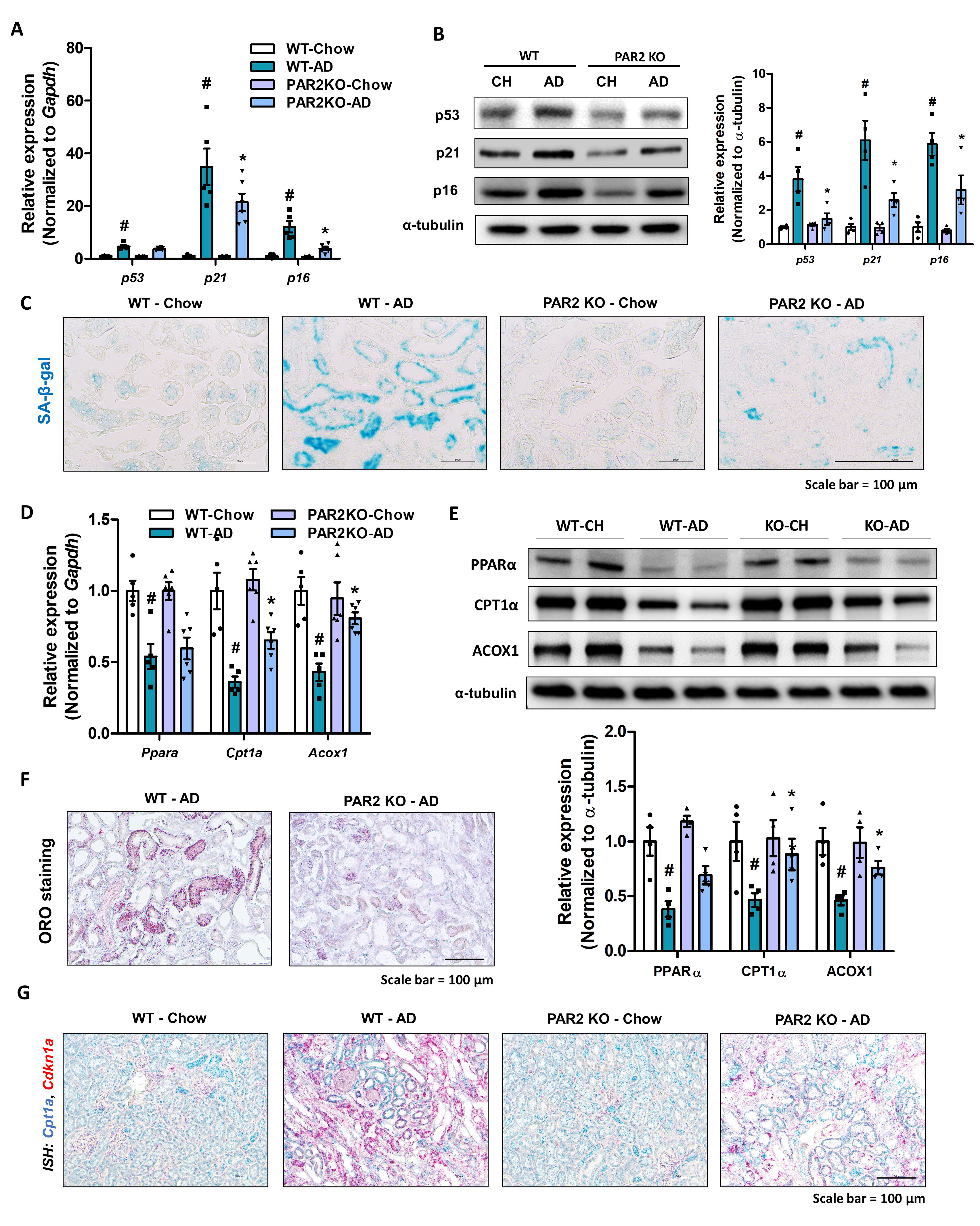

Supplement: Supplementary file 1 — Appendix S1. [file ACEL-23-e14184-s001.zip › S Figure 6 new.tif]

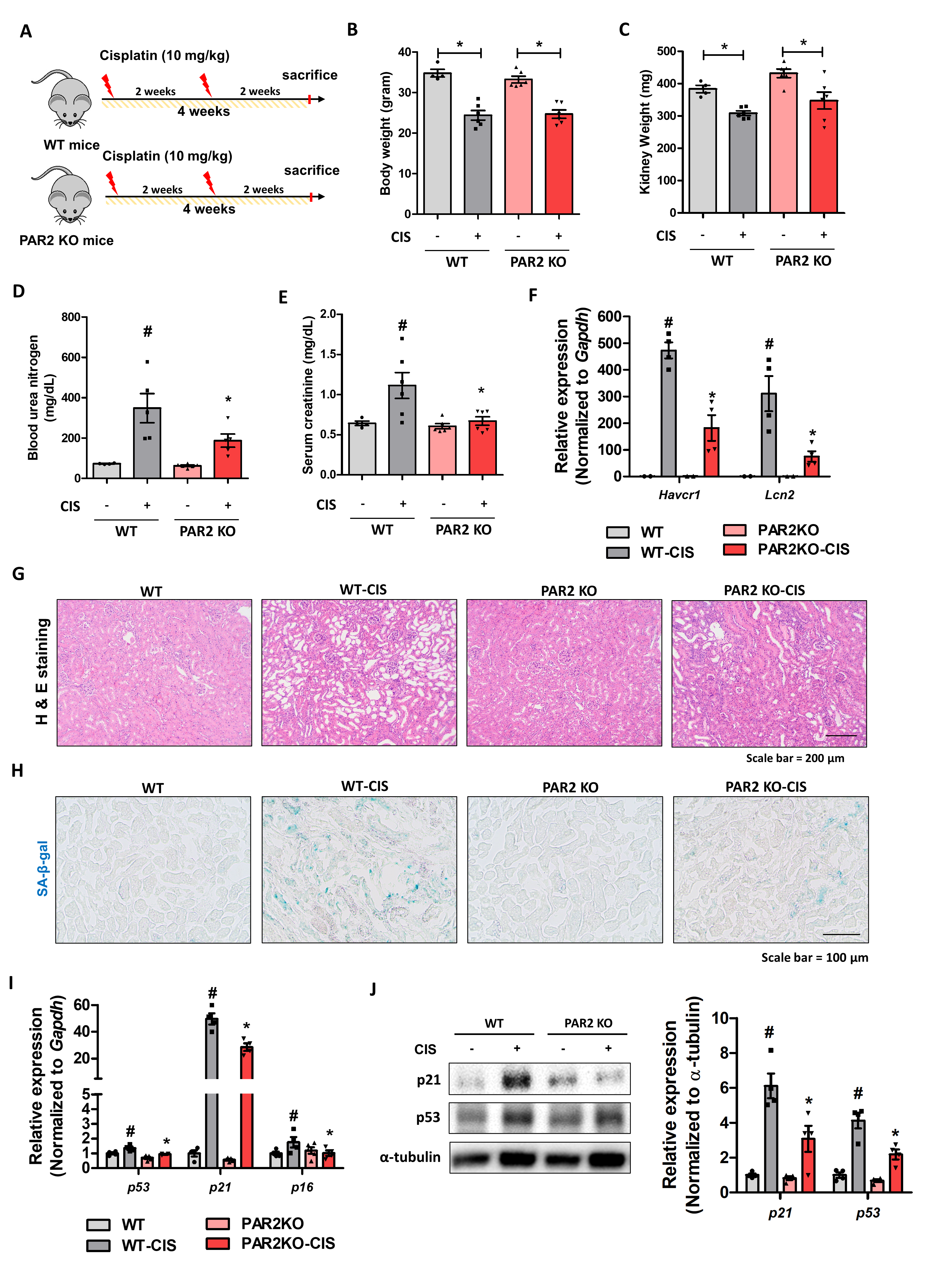

Supplement: Supplementary file 1 — Appendix S1. [file ACEL-23-e14184-s001.zip › S Figure 8 new.tif]

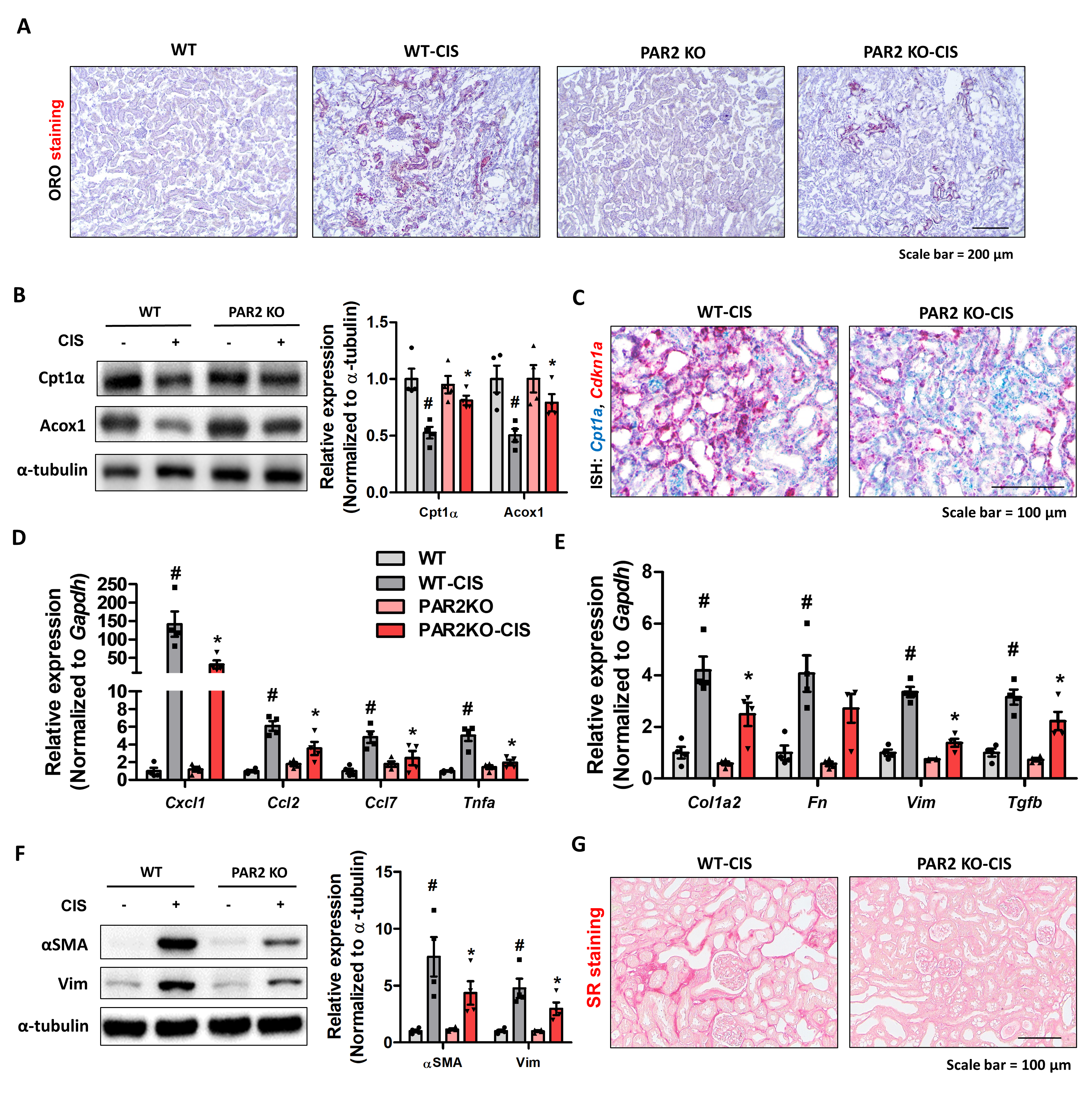

Supplement: Supplementary file 1 — Appendix S1. [file ACEL-23-e14184-s001.zip › S Figure 9 new.tif]

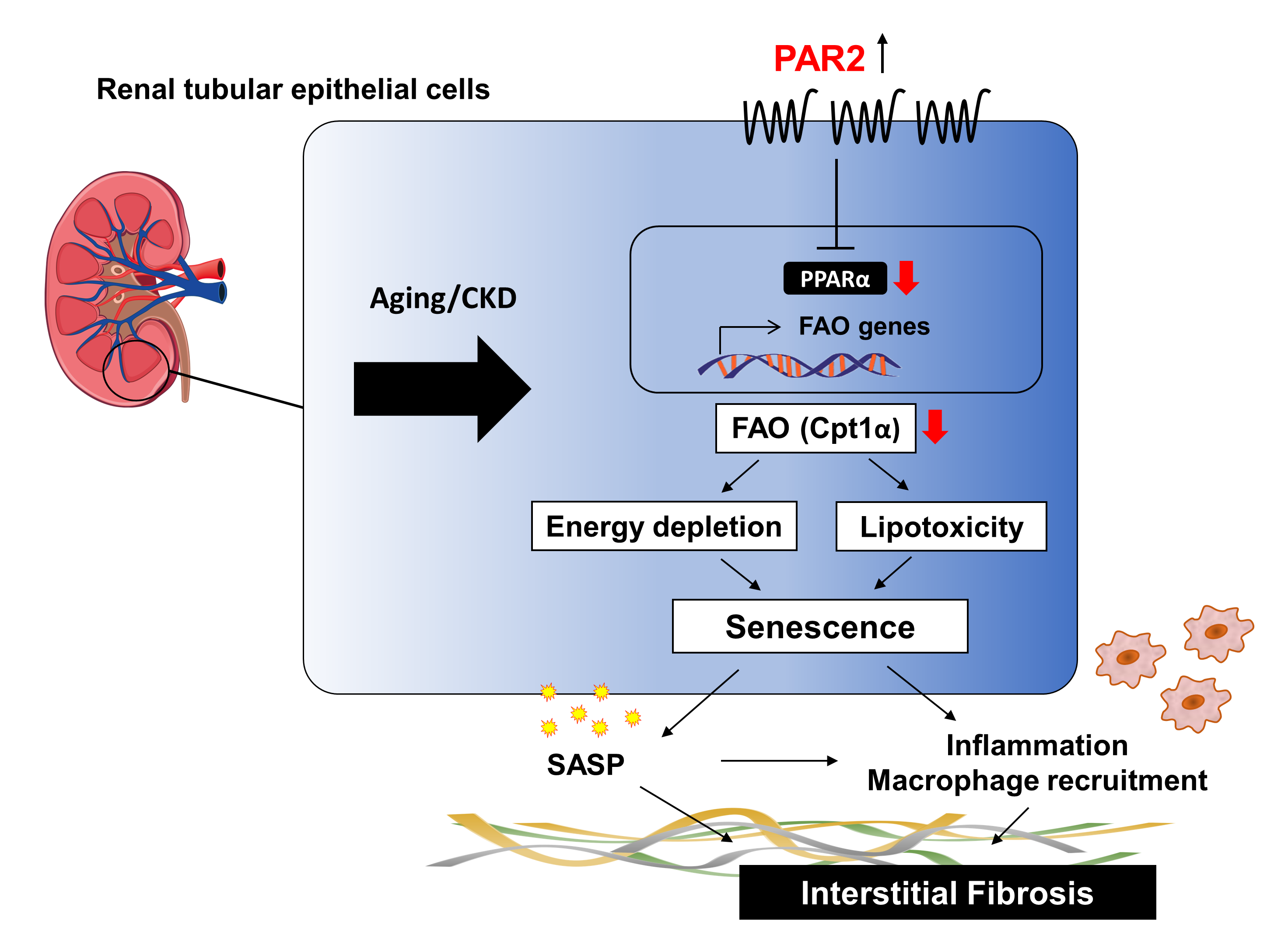

Supplement: Supplementary file 1 — Appendix S1. [file ACEL-23-e14184-s001.zip › S Figure10.tif]
